# A Comprehensive Review on Optical Properties of Polymer Electrolytes and Composites

**DOI:** 10.3390/ma13173675

**Published:** 2020-08-20

**Authors:** Shujahadeen B. Aziz, M. A. Brza, Muaffaq M. Nofal, Rebar T. Abdulwahid, Sarkawt A. Hussen, Ahang M. Hussein, Wrya O. Karim

**Affiliations:** 1 Advanced Polymeric Materials Research Lab., Department of Physics, College of Science, University of Sulaimani, Qlyasan Street, Sulaimani 46001, Kurdistan Regional Government, Iraq; rebar.abdulwahid@univsul.edu.iq (R.T.A.); sarkawt.hussen@univsul.edu.iq (S.A.H.); ahang.hussein@univsul.edu.iq (A.M.H.); 2 Department of Civil Engineering, College of Engineering, Komar University of Science and Technology, Sulaimani 46001, Kurdistan Regional Government, Iraq; 3 Manufacturing and Material Engineering, Faculty of Engineering, International Islamic University of Malaysia, Kuala Lumpur, Gombak 53100, Malaysia; mohamad.brza@gmail.com; 4 Department of Mathematics and General Sciences, Prince Sultan University, P.O. Box 66833, Riyadh 11586, Saudi Arabia; muaffaqnofal@gmail.com; 5 Department of Physics, College of Education, University of Sulaimani, Kurdistan Regional Government, Old Campus, Sulaimani 46001, Iraq; 6 Department of Chemistry, College of Science, University of Sulaimani, Qlyasan Street, Sulaimani 46001, Kurdistan Regional Government, Iraq; wrya.karim@univsul.edu.iq

**Keywords:** polymer electrolyte, polymer composite, nanocomposite, optical properties, taucs model, optical dielectric loss

## Abstract

Polymer electrolytes and composites have prevailed in the high performance and mobile marketplace during recent years. Polymer-based solid electrolytes possess the benefits of low flammability, excellent flexibility, good thermal stability, as well as higher safety. Several researchers have paid attention to the optical properties of polymer electrolytes and their composites. In the present review paper, first, the characteristics, fundamentals, advantages and principles of various types of polymer electrolytes were discussed. Afterward, the characteristics and performance of various polymer hosts on the basis of specific essential and newly published works were described. New developments in various approaches to investigate the optical properties of polymer electrolytes were emphasized. The last part of the review devoted to the optical band gap study using two methods: Tauc’s model and optical dielectric loss parameter. Based on recently published literature sufficient quantum mechanical backgrounds were provided to support the applicability of the optical dielectric loss parameter for the band gap study. In this review paper, it was demonstrated that both Tauc’s model and optical dielectric loss should be studied to specify the type of electron transition and estimate the optical band gap accurately. Other parameters such as absorption coefficient, refractive index and optical dielectric constant were also explored.

## 1. Introduction

Polymer electrolytes (PEs) were introduced for the first time by Fenton et al. in 1973 [1], and their technological significance on large-scale applications came to market in early 1980 [2]. Over the past three decades, a major focus on developing new PEs has been observed because of their wide uses in electrochemical supercapacitors, storage and conversion systems [3,4,5,6,7]. Polymer electrolytes (PEs) are membranes that consist of incorporated dissolved salts in polymer matrices (high molecular weight) [8]. These almost solid without solvent systems possess ionic conduction property; therefore, they have extensively been used in a number of electrochemical devices, for example, rechargeable batteries, solid-state batteries and especially lithium ion batteries. Recently, PEs possess additional potential uses particularly electric devices as well as electrochemical devices, for instance, electronic and electrochromic devices, rechargeable batteries, electric double-layer capacitors (EDLC), fuel cells, analog memory devices, dye-sensitized solar cells (DSSC), electrochromic windows and electrochemical sensors [3,4,5,6,7].

Previous studies revealed that various types of polymer electrolytes that possess high DC conductivity and show amorphous structure are preferable materials for rechargeable batteries applications due to their leakage free and low coast [9,10]. Based on recent advances, polymer electrolytes are crucial to be considered as novel and safe candidate materials for electrochemical device applications and to replace lithium ion batteries [10,11,12]. Recent studies indicated that PEs are essential for DSSC applications. O’Regan and Gratzel in 1991 developed a new type of solar cell called dye-sensitized solar cell DSSC, and due to unique properties, it became a new source of renewable energy [13]. An electrolyte in a DSSC plays an important role since the charge transport between the electrodes occurs through it [14]. Generally, electrolytes are classified into several types such as liquid, quasi-solid-state and solid-state electrolytes. Owing to the crucial impact on the efficiency of the solar cell, electrolytes were subjected to extensive studies [13,14,15,16,17,18,19]. The dry polymer electrolytes are often known as solvent-free electrolytes. It is also shown that both positively (cations) and negatively (anions) charged ions are loosely bound, therefore, can be movable. Generally, poly (ethylene oxide) (PEO) can be used as solvent-free polymer electrolytes with a relatively high molecular weight [20].

Unfortunately, the relatively high degree of crystallinity of PEO-based electrolytes at ambient temperature has exhibited a poor ionic conductivity [20]. To enhance ionic conductivity of polymer electrolytes, several methodologies have been implemented to reduce crystallinity; such as copolymerization, plasticization and blending via salt insertion, blending with other polymers and adding fillers/additives, respectively [21]. To be applicable, the polymer electrolyte has to fulfill several characteristics, especially adequate ion conductivity in the range between 10^−4^ and 10^−5^ S·cm^−1^ and insignificant electron conductivity under 10^−12^ S·cm^−1^ [12]. Additionally, high mechanical and electrochemical durability with reasonable optical characteristics (persisting in the contradiction of long-term UV irradiation, translucency or transparency) are necessary [22]. It is worth mentioning that the polymer hosts should possess several essential characteristic features including; polarity, a low steric hindrance to bond rotation and effective electron donors to form coordination bonds with cations. There are several superiorities of polymer electrolytes over traditional liquid electrolytes; phase-based ionic conductivity, transparency, solvent-free, flexibility and tunability, wide electrochemical stability, high ability to form a thin film, facile processability, relatively high ionic conductivity and lightweight.

**Figure 1:**
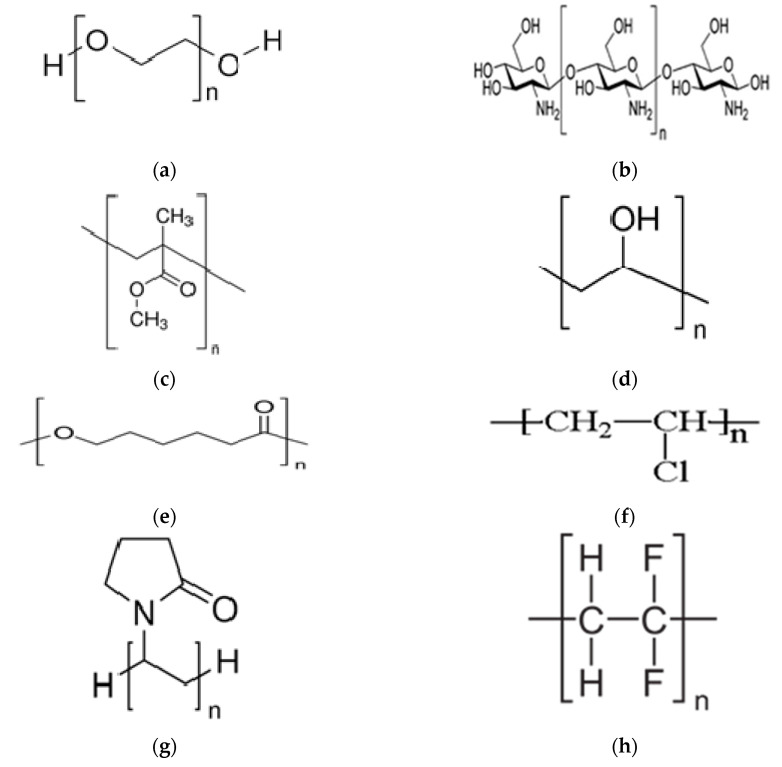
Various commonly used polar polymers structure in polymers electrolyte: (**a**) polyethylene oxide (PEO); (**b**) chitosan (CS); (**c**) poly methyl methacrylate (PMMA); (**d**) poly vinyl alcohol (PVA); (**e**) poly ξ-caprolactone (PCL); (**f**) poly vinyl chloride (PVC); (**g**) poly vinylpyrrolidone (PVP); (**h**) poly vinylidene fluoride (PVDF) [26,27].

The ionic conducting property of these electrolytes facilitates ionic species transportation which is comparable to certain ionic liquid electrolytes [23]. It is also impressive to mention that PEs are safe as they inhibit several issues, for instance, internal shortening, electrolyte leakage, corrosive character, harmful gas and noncombustible reaction products on the electrode surface [23,24]. Moreover, the polymer hosts should have some basic characteristic features; for example, it must be a polar polymer with low hindrance to bond rotation, and exhibit high power of an adequate electron provider to create coordination with cations, as shown in Figure 1.

The synthetic and natural polymer-based electrolytes have been increasingly focused in terms of electrical and optical properties because of legibility to be utilized in batteries, fuel cells and capacitors [25,26,27,28]. The interest in dealing with the nature of the light interaction with such new materials increases, especially changing from solar cells to light-emitting devices. Moreover, those polymers that possess unique optical properties to encourage many research groups to take into consideration these polymers to be utilized in optical devices, sensors and LEDs as well. To change the optical properties of certain polymers, it is viable to tune these properties using dopant materials. Two factors that govern the new identity of polymer systems are the type of dopants and their concentrations [29]. For example, the addition of metals, semiconductor particles, slats and metal complexes can manipulate both optical and electrical properties [21,30,31,32,33].

To be applicable in certain applications, the optical properties of polymer electrolytes, for instance, absorption edge, transmittance, reflectance, energy gaps, dielectric loss (ε”), dielectric constants (ε’) and extinction coefficient (K) have to be measured. 

The aim of this review is directed to show a comprehensive understanding of the optical properties of polymer electrolytes and composites. Determination of the types of electron transitions accurately using the ε” parameter is also focused. Based on both theoretical and experimental principles, it is reached that the ε” parameter is effective in specifying the types of electron transitions with the aid of Tauc’s model. The categorization of polymers and their composites is clearly shown. Ultimately, deep insight into band gap energy is provided for polymer electrolytes and their corresponding composites.

## 2. Solid Polymer Electrolytes and Their Composites

### 2.1. Polymer—Salt Complex and Dry Solid Polymer Electrolytes (SPEs)

**Figure 2:**
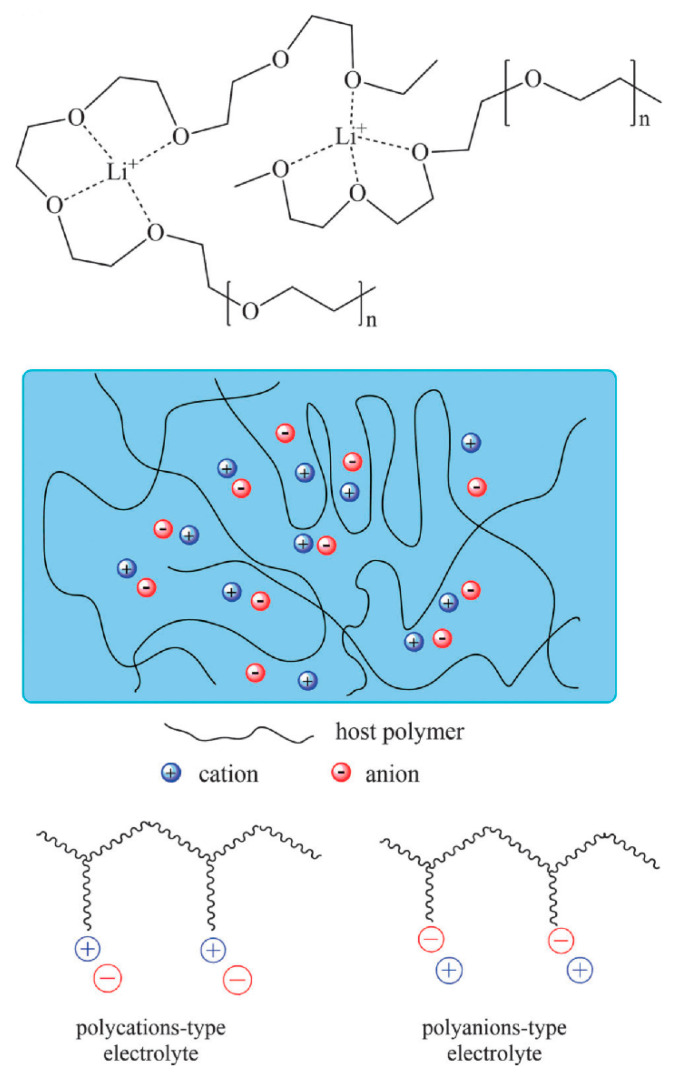
Dry solid polymer electrolytes PEO with Li^+^ salt structure [35].

To prepare dry SPEs, it is necessary to make a complexation by dissolving ionic inorganic salts in coordinating polar polymer hosts [23,34]. The desired dry SPEs are obtainable by a mainstream using polymers with large molecular weight, such as poly (ethylene oxide) (PEO) or poly (propylene oxide) (PPO) that are complexed in various Li^+^ ion salts. Different lithium salts can be used in the complexation with PEO to prepare SPE membranes, for example, LiX (where X = I, Cl, Br, ClO_4_, CF_3_SO_3_, BF_4_, AsF_6_, etc.) [35]. The dry solid polymer electrolyte PEO was interacted with Li^+^ salt and presented in Figure 2. It is vital mentioning that the sequential repetition of oxyethylene group; –CH_2_–CH_2_–O–, and the existence of polar groups; –O–, –H–, –C–H–, in the chains of polymer PEO/PPO composite strengthened its ability to dissolve, followed by complexation of the ionic salts [23,35]. The extent of the polymer–salt complex is managed by competition among polymers lattice energies and inorganic salts on one side and the solvation on the other side [36]. Herein, this focus on the optical properties of polymers has been explained on the basis of these wide applications in optical devices [37,38]. The purpose of directing the optical properties in this survey is to reach optimum reflection, antireflection, interference and polarization properties [37]. This is achievable if there is a modification in the optical properties implemented using the dopant incorporation process [37]. This incorporation of dopant into polymer matrices results in changing chemical composition; in other words, increasing in optical absorption results from changing the power of polarization, identity of the modifier, coordination number and the number of nonbonded oxygen functional groups within the polymer bodies [39]. The optical absorption changes, mainly the absorption edge shifts and shapes are two promising methodologies in dealing with comprehending the fundamental process of optically induced transitions in noncrystalline and crystalline materials. This offers a better insight into the structure of the energy band. To achieve higher efficiency of photonic and optoelectronic devices, considerable development has been obtained in comprehending the essential chemical and physical characteristics of polymers. However, establishing a correlation between these two properties is not achieved yet. Characterizations of optical properties of polymers, for instance, infrared dichroism, optical absorption, Raman polarization and luminescence spectra, are among the crucial techniques to examine the electronic properties [40,41,42].

These techniques are based on the compromise between the highest occupied molecular orbital (HOMO) and the lowest unoccupied molecular orbital (LUMO) for electron transition. To increase the performance of optoelectronic and photonic devices, modifications have to be carried out via blending, functionalization of the polymer, cross-linking, copolymerization, hybridization and grafting with other inorganic polymers [43]. To gain a new polymer electrolyte structure, the medication involves a combination of PEO main chains with block copolymers, grafted polymers and cross-linked polymer networks [35,36,44]. This modification resulted in a lesser degree of crystallinity and a small glass transition temperature T_g_ of polymer electrolytes [35,45].

### 2.2. Composite Polymer Electrolytes

There are dissimilar kinds of nanofillers (NFs), mixing of inorganic nanoparticles (NPs) that are incorporated into polymer matrices, resulting in nanocomposites (NCs) of polymer and inorganic. This is of technological importance owing to their prospective uses as magneto-optic and optoelectronic devices [46].

The most commonly used inorganic NFs in the corporation process are semiconductors, for instance, Ge, PbS, CdS and metal oxides (ZnO and TiO_2_) as well as metals (Ag and Cu) and metal alloys (Co/Pt and Au/Pt). It is worth mentioning that this enhancement in optical properties of polymers opens the opportunity to be utilized in color filters, polarizers, light-emitting diodes, optical communication devices, data storage devices and in the biomedical areas as well. In the next sections, there will be a review of the optical properties of metal NPs, metal complexes, semiconductor NPs, ceramic NPs, carbon-based materials and polymer composites [47].

The word composite polymer electrolyte involves the dispersion of nano/microsized fillers in solid polymer electrolytes (SPEs). For example, relatively high conducting zeolites, solid superacid sulfated-zirconia and ionites have been used as fillers. In addition to all these, Al_2_O_3_, SiO_2_, TiO_2_, etc., are often used.

**Figure 3:**
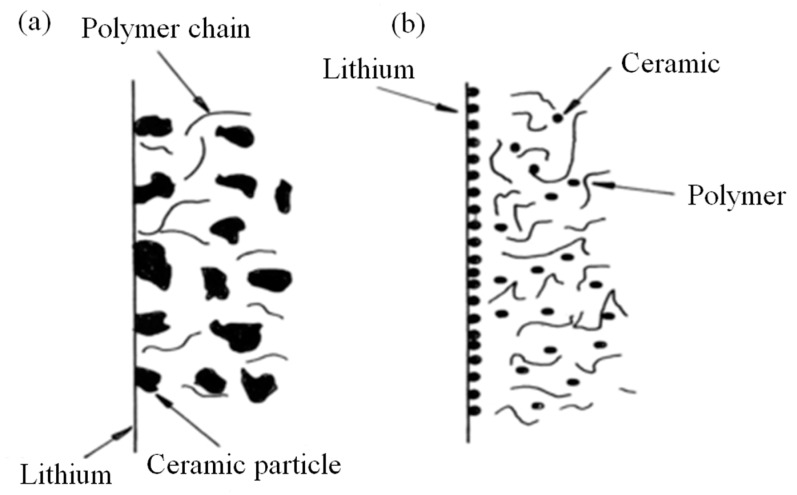
Illustration diagram of polymer host, nano/micrometer-sized inorganic doper within the polymer with particle radius: (**a**) micrometer; (**b**) nanometer [53].

It is critical to notice that the radius and the physical behavior of particle dispersion play a vital role. Hence, the dispersal nanosized dopants were determined to be efficient in the composite SPE systems, in particular on the basis of these enhancements in the mechanical, physical, electrochemical and optical characteristics. This new category of materials was coined to as NC polymer electrolytes (NCPEs) [48,49,50,51,52]. The diagram illustration of polymer, nano/micrometer is presented in Figure 3.

There are two main challenges facing NC preparation: homogeneity and uniformity of dispersion within the polymer matrices [54,55]. In general, two factors; the polymer matrices and the extent of the complexation between the polymer matrices and NFs govern the success of NC preparation. The influence of solvents during polymer dissolution is inevitable [56]. Moreover, it is well-known that the surface area of the NPs materials has relatively large surface energy, so that there are aggregation and agglomeration possibilities. These issues are clearly observed throughout the literature [46,54,57] in which attempts have been directed to achieve a satisfactory distribution of inorganic NPs within the polymer matrices. Therefore, different methodologies have been proposed in an attempt to avoid aggregation of the NFs during the incorporation process, such as independent preparation of the NPs and then mixed with the solute of monomer followed by polymerization in the sitting process [58,59]. Alternatively, dispersion and dissolving of the NPs and polymer, respectively in the same solvent separately can be carried out. Moreover, the dispersion of the NPs in a solvent is different from that used for dissolving the polymer in which both are soluble in each other. This methodology needs the adjustment of NPs surface [59,60], in which organic carboxylic acids as modifiers can be used, for example, oleic acid [61]. It is also realized that the modification can be carried out by coating the NPs with polymers [62,63]. Another modification comprises ultrasound treatment where NPs aggregates can be broken down; however, the duration of sonication should be optimized concisely [63]. Finally, incorporation of the NPs was performed using precipitation within the polymer matrices in the form of bulk polymers or even monomers [57,58]. This procedure avoids NPs aggregation within the polymer body. Throughout the literature, it has been confirmed that the sonication is the most efficient way of breaking aggregated ZnO NPs. Thereby; the ZnO NPs cannot reaggregate after sonication because of the entrapment within the polymer [60].

These composites that are based on polymers have been focused by a number of researchers due to the interesting electrical, electronic and optical properties. The composite-based polymers have many applications, for instance, in angular acceleration accelerometers, electronic packaging, acoustic emission sensors, integrated decoupling capacitors [64,65], optoelectronic devices and organic solar cells [66]. The organic/inorganic composites are characterized by flexibility, isotropic property and amorphous phase. These materials have been investigated in terms of structure to be used in biomedical areas. In addition, the organic/inorganic composites can be used as membranes in fuel cells [67]. These mixtures of organic/inorganic materials outweigh the corresponding single organic and inorganic ones on the basis of properties, for instance, enhancement in electrochemical stability [68]. However, in certain composite electrolytes the additives, such as α-Al_2_O_3_ lithiumborosulfate glass and γ-LiAlO, were determined to possess a negligible or a tiny depressing impact on the conductivity of polymer electrolyte [53,69,70].

## 3. Optical Absorption of Polymer Electrolytes and Composites

### 3.1. Optical Absorption Spectra of Polymer Electrolytes

**Figure 4:**
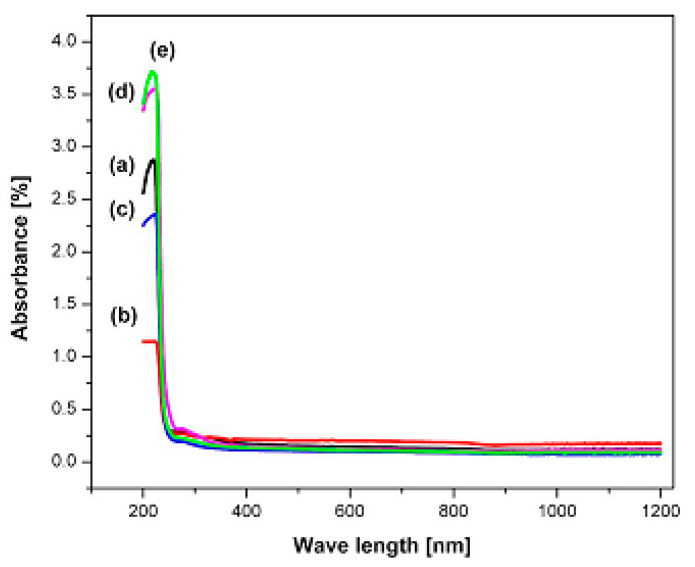
UV absorption spectra of PVA-LiPF6 films (**a**) 100:0; (**b**) 95:05; (**c**) 90:10; (**d**) 85:15; (**e**) 80:20 [76].

**Figure 5:**
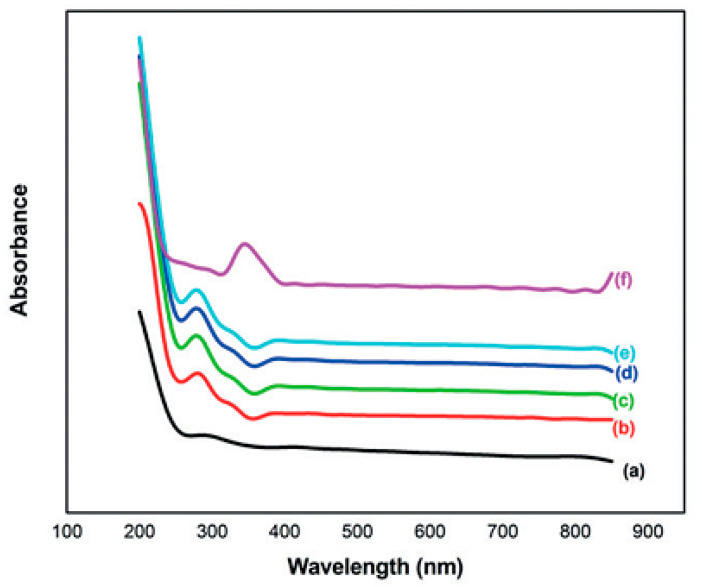
UV–vis absorption spectra of pure and Mn^2+^-doped PVC polymer films: (**a**) pure; (**b**) 1 mol %; (**c**) 2 mol %; (**d**) 3 mol %; (**e**) 4 mol %; (**f**) 5 mol % [77].

**Figure 6:**
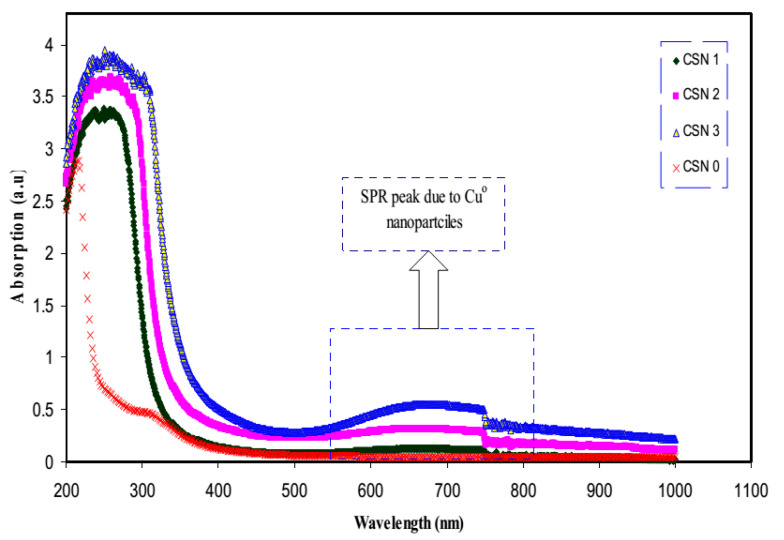
Absorption spectra of pure CS and CS/CuI solid electrolyte films. The surface plasmonic resonance (SPR) peak appearing at approximately 667 nm for CS/CuI samples is related to the existence of Cu metallic NPs [78].

**Figure 7:**
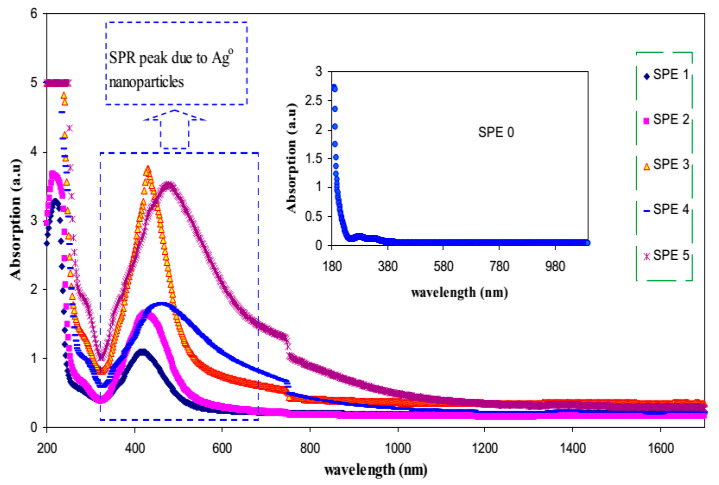
Absorption spectra of pure PVA (inset) and PVA/AgNt solid films. The SPR peak appearing at about 422 nm for PVA/AgNt samples is related to the existence of silver NPs [79].

The optical properties of polymer electrolytes have been studied intensively because of their wide applications in electronic and optical devices, such as chromic display and optoelectronic devices [25]. Recently, many laboratories have been supported by various companies in which large efforts are devoted to developing new materials to be used in photonic and optoelectronic devices, such as light-emitting diodes (LEDs), organic photovoltaic devices and polymer lasers [71]. From the literature, it is noted that UV–vis spectroscopy has been commonly used for optical and electronic characterizations of polymer electrolytes [25,72,73,74,75]. It is also observed that polymer electrolytes that are impregnated with alkali metal salts have exhibited unique optical absorption behavior. Figure 4 shows the optical properties of PVA-LiPF6 solid polymer electrolytes [76]. It can be seen that there is a shift in absorption to a higher wavelength upon the addition of 20 wt% of LiPF6. It was also documented that the polymer electrolyte film of PVC doped with MnSO_4_ undergoes shifting in the absorbance spectra to the higher wavelength as the concentration of MnSO_4_ is increased (see Figure 5) [77]. Aziz et al. recorded a significant shift within the absorption spectra of the chitosan-based electrolyte that incorporated copper and silver salts as presented in Figure 6 and Figure 7, respectively [78,79]. This shift in absorption has been correlated to a huge reduction of ions to metallic particles [78,79]. It was also associated with the effective irradiation of polymer electrolytes. Yesappa et al. has documented the effectiveness of irradiation on the absorption capacity (optical property) and electric performance (ionic conductivity) of PVDF-HFP/LiClO_4_ (90: 10 PHL10) electrolyte films [80]. It was observed that with increasing irradiation dose, the absorption shifting increases correspondingly [80].

### 3.2. Optical Absorption Spectra of Polymer Composites

#### 3.2.1. Metal Nanoparticles

It is well-studied that the nanoscale materials possess sole electronic and optical characteristics that are drastically different from their bulk states [81]. Here, it is shown that the nanostructures enable noble metals to be very sensitive optical nanosensors, surface-improved spectroscopies and components of photonics [82].

There are several methodologies that have been implemented in the synthesis of metal NPs, such as electron beam irradiation, polyol, in situ chemical synthesis, microemulsion, reverse micelles, wire explosion and green chemistry pathways. The judgment on the proper one relies on the tendency of metal ions to form complexes, for example, soluble polymers not only form complexes with metal ions, but also avoid NPs aggregation [83,84].

Despite various kinds of NFs, mixing of inorganic NPs with polymer matrices, creating NCs of polymer and inorganic [46] is of technological attention owing to their prospective utilizations as magneto-optic and optoelectronic devices. For example, the most employed inorganic NFs are metals (e.g., platinum (Pt), gold (Au), silver (Ag), iron (Fe), germanium (Ge) and copper (Cu)). To make an enhancement in the optical properties, these NFs are extensively applied in various polymers [47].

The term plasmon originates from free electrons within metals when exposed to electromagnetic radiation create surface plasmon resonance (SPR) absorption [47]. It is well-known that for precious metals, such as gold, silver and copper, the surface plasmon oscillation frequency lies within the optical absorption region. This causes the generation of a characteristic color in each metal at the nanoscale. It is of great importance to know that two factors determine this phenomenon; mainly by the shape and partly by the radius of the metal NPs [47].

Incorporation of spherical metal NPs into polymers allows modification in the physical properties of polymers and the creation of new features in the polymer matrices as well. It is confirmed by a number of research studies that the incorporation of metal NPs into polymer electrolytes resulted in the improvement of the optical properties [78,85].

Copper (Cu) is one of the widely utilized metals in electrical and electronic usages because it possesses a relatively large conductivity and cost-effectiveness. Miniaturization of devices, such as sensing and electronic ones results in different characteristics in terms of electronic, photonic, chemical and biological aspects [82]. It is also worth mentioning that this wide usage of copper NPs originated from the beneficial characteristics of this metal, such as satisfactory thermal conductivities. This property of copper is exploited to be used in cooling fluids for conductive inks and electronic systems [86]. Another usage of this precious metal includes its incorporation into or “supported on” another metal and then mostly employed in various catalyst processes and NCs fabrications with unusual electrical, optical and magnetic characteristics [87].

Three interesting physical properties of copper NPs; which are a relatively high melting point, small electrochemical movement behavior and excellent ability to solder have attracted particular attention of research groups to focus on [88]. Therefore, copper replaces other metals for usage in heat transfer and inkjet printing [89]. One of the studies showed that the greatest capable technology that employs precious metals, for example, silver and gold for printing greatly conductive elements is called inkjet printing. Therefore, copper is often preferred in coating a substrate of plastic through inkjet printing to fabricate highly conductive copper patterns since it is cost-effective and has a relatively large conductivity [90].

**Figure 8:**
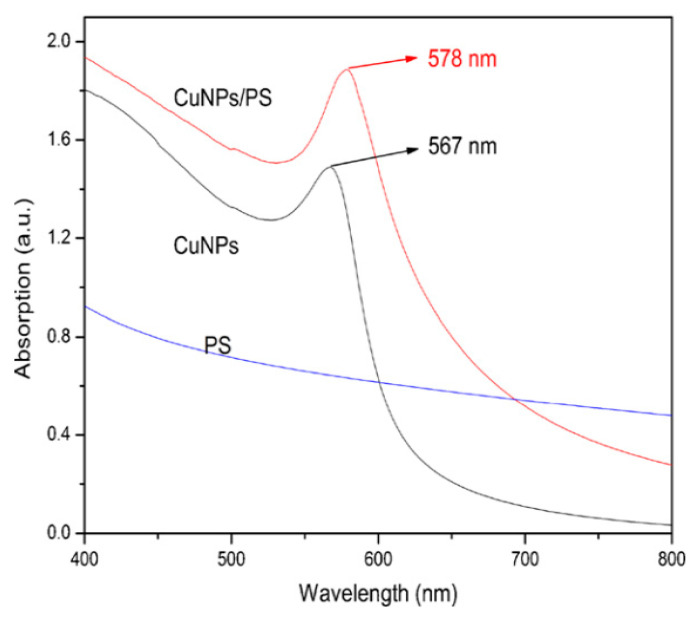
UV–vis spectra of PS colloid, CuNPs colloid and CuNPs/PS colloid [91].

Tian et al. [91] synthesized copper NPs (CuNPs) colloid using copper sulfate and hydrazine hydrate as a precursor and reducing agent, respectively in the presence of cetyltrimethylammonium bromide (CTAB) and poly(N-vinylpyrrolidone) (PVP). This is a copper NPs/polystyrene (CuNPs/PS) NCs via emulsion polymerization in situ. Figure 8 exhibits the UV–vis spectra of CuNPs colloid, PS colloid and CuNPs/PS composite colloid. It is interesting to notice that the absorption band is located between 500 and 600 nm is a characteristic of CuNPs colloid as a result of surface plasmon resonance impact [92,93]. Based on Mie theory [94], the surface plasmon resonance peak position is correlated to the morphology of the surface of metal nanostructures and copper NPs with a spherical shape that possesses merely a single peak of surface plasmon resonance. The single unique robust surface plasmon resonance absorption peaked at 567 nm in the UV–vis spectrum of CuNPs colloid (Figure 8), suggesting that the CuNPs have to be spherical [92,93]. The position of the surface plasmon resonance peak of CuNPs shifted from 567 nm (CuNPs colloid) to 578 nm (CuNPs/PS composite colloid); in other words, a redshift occurs. This can be associated with the introduction of polystyrene to the NPs surface bodies in the form of a uniform coating on the copper NPs surface. In a kinetic study, the CuNPs colloid UV–vis spectra were recorded over various time intervals. It was observed that no noticeable change in the spectra of UV–vis occurred after a couple of weeks, indicating that the CuNPs colloid possesses sufficient stability. 

Tian et al. [91] explained that the cause of broadness of the absorption peak was related to the size distribution of the NPs. This was in accordance with TEM observations in another work [95]. It is evidenced that the shape of CuNPs is spherical which is in accordance with that obtained from the UV–vis spectrum [95]. It was noted that the CuNPs disperse uniformly. This is due to micelle formation during the NPs synthesis of copper in which aggregation is exhibited. The data analysis has shown that the average particle size was in the range of 10 to 20 nm [91].

**Figure 9:**
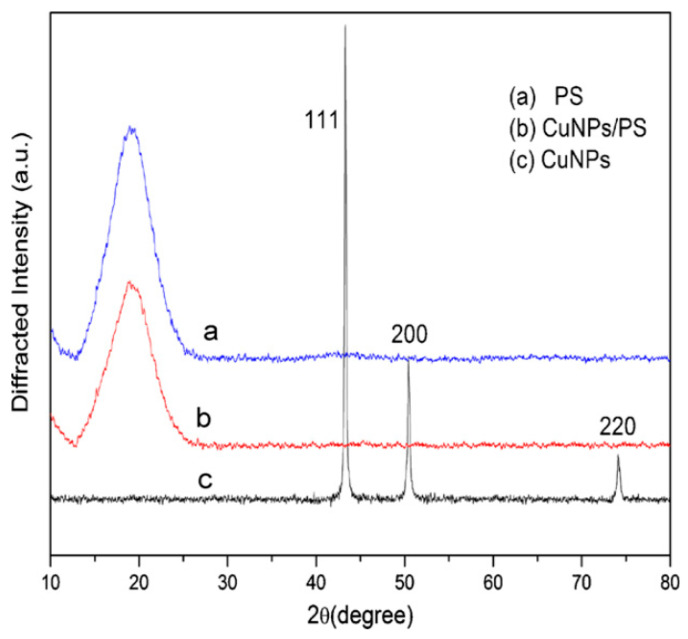
XRD patterns of PS, CuNPs and CuNPs/PS composites [91].

The TEM results confirmed that the copper NPs are spherical and single crystals [95]. The XRD analysis emphasizes the results obtained from the TEM [95]. Tian et al. [91] presented the XRD patterns of PS (a), CuNPs/PS composite (b) and CuNPs (c) in Figure 9. In Pattern C, the main feature diffraction peaks are located at 2θ = 74.2°, 50.4°, and 43.3° equivalent to the (2 2 0), (2 0 0), and (1 1 1) crystal faces of copper, respectively. The wide typical diffraction peak of polystyrene peaked at 2θ = 19.2° in both a and b patterns. The coating of polystyrene on the surface of the CuNPs rationalized the disappearance of the typical CuNPs diffraction peaks in pattern b.

In another study, synthesis of CuNPs was carried out via a hydrothermal process using copper chloride as a source of ions and carboxymethyl gum kondagogu (CMGK) as a reducing agent [95]. In the study, XRD was used to prove the crystalline structure of the NPs and the peak broadening is mainly related to the size of the NPs [96]. The analysis of XRD was exploited to calculate the size of the NPs using Scherer’s formula and found to be around13.8 nm. From both TEM and XRD, the calculated particle size is consistent that 14 ± 2 nm obtained [95].

**Figure 10:**
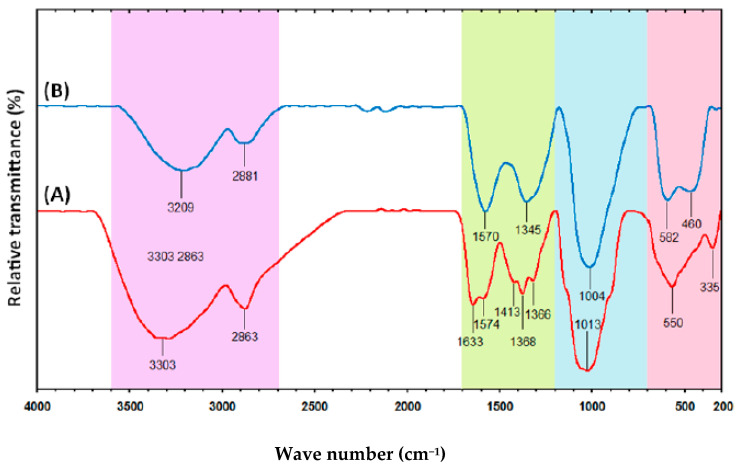
(**A**) Pure chitosan FTIR spectrum and (**B**) fabricated Cu-NPs in chitosan [97].

Usman et al. [97] used chitosan (CS) media in CuNPs fabrication via a chemical reaction methodology. The synthesis of the NPs in an aqueous solution has been carried out in the existence of SC and CuSO_4_·5H_2_O as a precursor and stabilizer, respectively. The complexation among the chitosan and the fabricated NPs by Fourier transform infrared (FTIR) spectroscopy was investigated and demonstrated the capping of the NPs by Cts. As evidence, C=O vibration band is located at 1633 cm^−1^ corresponds to the existence of an amide group in the Cts spectrum in Figure 10 [98].

In the spectrum, it is shown that transmissions at 3303 cm^−1^ are attributed to the overlap of stretching vibrations of O−H and N−H [97].

It is seen that the peak lies at 2863 cm^−1^ is related to the C−H stretching mode and the peaks at 1633 and 1574 cm^−1^ are correlated to –NH_2_ bending. The other peaks at 1413, 1368 and 1366 cm^−1^ result from C−H bending and 1013 cm^−1^ to −C−O skeletal stretching. In a similar way, the same result was obtained for CS mediated CuNPs (Figure 10B). It was also noted that slight moving in the band occurs toward the blue shift (from 3303 to 3209, 1574 to 1570, 1368 to 1345 and 1013 to 1004 cm^−1^) [98]. An interesting observation is that a new peak appears at 460 cm^−1^ that can strongly be related to the bond vibration as a consequence of the complexation between the CuNPs and the CS host. This indicates a relatively strong interaction between the CuNPs surface and the Cts amino and hydroxyl groups, suggesting the ability of the polymer to cap the NPs [86].

Habibi et al. [99] used thermal reduction in the synthesis of CuNPs. The CuNPs prepared from bis-(oxalato)-copper(II) as a precursor and various capping agents with different radii ranging between 8 and 20 nm, respectively. The SEM analysis revealed a relatively large surface free energy of the bare CuNPs, which increased a tendency to agglomeration. 

It is informative to use UV–vis spectroscopy as a powerful technique in the characterizations of NF in the polymer matrices from metal NPs, especially to estimate the size, dispersion, stability of the NCs and the interactions [47].

Another type of NPs which is under extensive study is silver NPs (AgNPs). These NPs possess vital and fascinating properties that are applicable in biomedical fields. Silver NPs are characterized due to exceptional thermal, electrical and optical characteristics that are doped into various products ranging from photovoltaics to chemical and biological sensors. The utilization of silver NPs is increasingly popular in different sensors and products due to the interesting optical properties.

**Figure 11:**
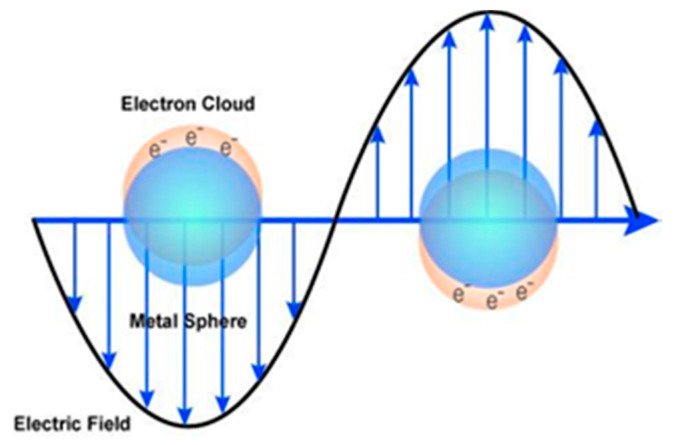
Surface plasmon resonance while the free electrons in the metal nanoparticles (NPs) derive into oscillation because of a robust interaction with incident light at a certain wavelength [102].

Furthermore, these NPs are characterized by an exceptional ability to scatter and absorb light and are different from the dyes and pigments. These characteristics are determined by the shape and the radius of the NPs. There is a relatively strong interaction between the silver NPs and the light that causes enrichment in the conduction electron on the metal surface. This results in a successive collective oscillation and the excitation come from light absorption at certain wavelengths (Figure 11). In fact, based on the surface plasmon resonance (SPR), the oscillation leads to high absorption and scattering. Therefore, a relatively high extinction coefficient has been counted for these NPs for the processes of absorption and scattering cross-section that is 10 times greater than the physical cross-section [100,101].

Khanna et al. have employed two various reducing agents known as hydrazine hydrate (HH) and sodium formaldehyde sulfoxylate (SFS) in dispersing Ag NPs into poly(vinylalcohol) (PVA) through silver reduction in situ [103].

To evaluate the quality of silver NPs via using two different reducing agents, UV–vis spectroscopy is helpful. The characteristic SPR peaks of the silver NPs using HH and SFS in the PVA centers at 418 nm. The sharp feature of the peaks indicates the small size NPs production and uniform distribution throughout the matrix. Interestingly, the peak is rather wider when HH is employed like the reducing agent that demonstrates the uneven distribution of particle size. Further analysis showed that the peak width at half the maxima (PWHM) is a more informative parameter in the UV–vis spectroscopy. Their work provided a good insight into the distribution of particles and their sizes within both the medium and matrix. On the one hand, the analysis of PWHM for the synthesized Ag NPs by HH was documented to be roughly 110 nm [103].

It is interesting to note that the PWHM of the synthesized NPs using SFS is only 85 nm. Thus, UV–vis spectra analysis is a powerful technique to tackle the size and distribution of the NPs in the polymer matrix. If the high-quality and uniform distribution are taken as criteria, the SFS gives better results than HH [103]. Previously, it has been confirmed that solution cast methodology is facile and effective in the preparation of polymer composite-based on polyethylene oxide (PEO) [104]. Nowadays, this methodology has been implemented for the preparation of silver nanoparticles because it obeys green chemistry principles. A peak appeared around 320–780 nm for the polymer composite [104], verifying the existence of the Ag NPs within the polymer matrix. Another characterization has to be performed dealing with the stability of the synthesized colloidal solution of NCs by means of UV–vis spectroscopy. This can be useful by noticing the SPR absorption peak of synthesized Ag NPs. At first glance, the stability of the Ag NPs was carried out after 15 months of storage using UV–vis absorption spectra in ref. [101]. The absorption band around 320–780 nm resulted owing to the Ag NPs surface plasmon absorption has not shown any change in both cases, demonstrating the relatively large stability of silver NPs within the host polymer.

**Figure 12:**
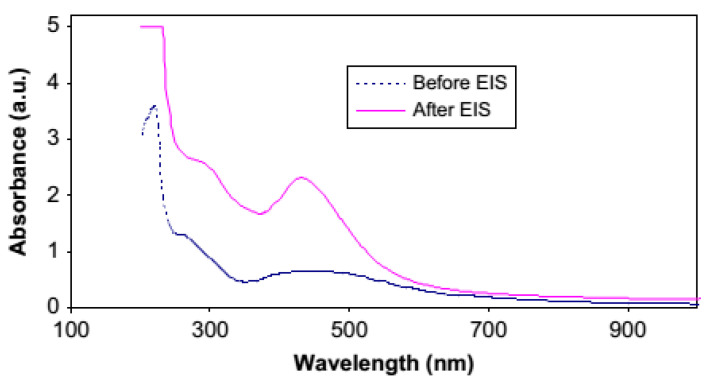
UV–vis spectra of chitosan–AgCF_3_SO_3_ before (room temperature) and after (high temperature) electrochemical impedance spectroscopy (EIS) [105].

Azizet al. studied silver ion (Ag^+^) reduction to the silver nanoparticle (Ag^o^) in chitosan: AgCF_3_SO_3_-based solid polymer electrolytes and characterized via UV–vis and XRD spectroscopies [105]. It is observed that the SPR and crystalline peak intensity are effectively impacted by temperature changing (see Figure 12). In another work, it was shown that there is a high possibility of silver ion (Ag^+^) to silver NPs (Ag^o^) within polymer electrolytes enriched with functional groups [79]. Furthermore, the effect of the dielectric constant of the host polymer on the shape and intensity of SPR was revealed [79].

Another interesting NPs are those from gold that have also been synthesized and utilized on a large scale because of the exciting colors developed due to their reaction with optical light. To date, the sole optoelectronic characteristics were examined and exploited in the industrial uses, for instance, therapeutic agents, electronic conductors, organic photovoltaics, sensory probes, drug delivery in the area of biological and catalysis. 

Recently, there are intensive studies on electronic and optical characteristics of gold NPs via manipulating the surface chemistry, aggregation state, shape and size. For example, the interaction of light with gold NPs is governed by several factors: Environment, physical dimension and size. In an explanation, when a light oscillates at an electric field and propagates near colloidal NPs results in interaction with electrons at certain stages [47,106].

#### 3.2.2. Metal/Ligand Complexes

It is apparent that a great risk is posed by heavy metals, particularly when released into the environment as pollutants worldwide [107]. It is well-known that the atomic mass of heavy metals are in the range of 63.5 to 200.6 u with a specific gravity of more than 5.0 [108].

A group of metals and metalloids has been used in many applications on the basis of their high atomic density (4000 kg m^−3^) [109]. These metals have played vital roles in the field of chemistry in which new materials can be developed that serve as reagents and catalysts in both fundamental and applied areas [110]. However, several treatments have been established to eliminate heavy or trace metals, for instance, precipitation, neutralization, biosorption, ion exchange and adsorption. These technological techniques have been extensively implemented in wastewater treatments [111]. Among these, adsorption technology is considered to have a satisfactory effect and cost-efficient for treating heavy metal wastewater. This way is characterized by flexibility in terms of design, operation and high-quality treated effluent [108]. From an economic and technological viewpoint, for industrial processing to be competitive, it is essential to establish and execute cost-effective procedures for the elimination/retrieval of metals [112]. Indeed, the green chemistry technique is one of the effective and efficient ways. It is impressive to extract sufficient functional groups and polyphenols from tea solutions. These extracts are able to capture the cations of heavy metals and synthesize metal complexes.

Polyphenols consist of amino acids and alkaloids, avins, catechins, isomers of avins and other constituents of tea. The studies have also provided the most evident chemical or molecular structures of the constituents of black tea [113,114]. Dryan et al. [114] recently documented that polyphenol compounds are largely prevalent in an aqueous mixture of black tea. It is also shown that the major tea components are polyphenols, polyphenol conjugates and polymerized phenolic structures. Furthermore, the three types of tea, such as black, white and green tea contain a range of mixture of conjugated flavonoids [115].

In the earlier work, it has been confirmed that the extraction of the contents in black and green tea solution is useful. The contents play an impressive role in reducing the band gap energy of polar polymers, for instance, PVA and PMMA [66,116]. From the FTIR study, it has been emphasized that polyphenol, hydroxyl group and carboxylic acid groups exist in both types of tea [116]. Based on the experimental findings, active functional groups act as ligands that have the ability to form complexes with both polymers and transition metals. For example, the polyphenols obtained from tea have been used in the complexation with metal cations that evidenced from electron paramagnetic resonance (EPR) technique [117]. From physics and industry perspectives, metal complexes with improved absorption behavior in the visible region, in other words, relatively high molar absorptivity (ε) is extremely important, especially in photonics, solar cells and optoelectronics devices. Brza et al. [118] synthesized copper (II)-complex using the green chemistry-based method. Taking advantage of the extracted tea solution, copper (II)-complexes can be formed successfully. The tea solution enriches in OH and NH functional groups, polyphenols and conjugated double that have the ability to catch the cation of heavy metals such as copper, cadmium, cerium, etc.

**Figure 13:**
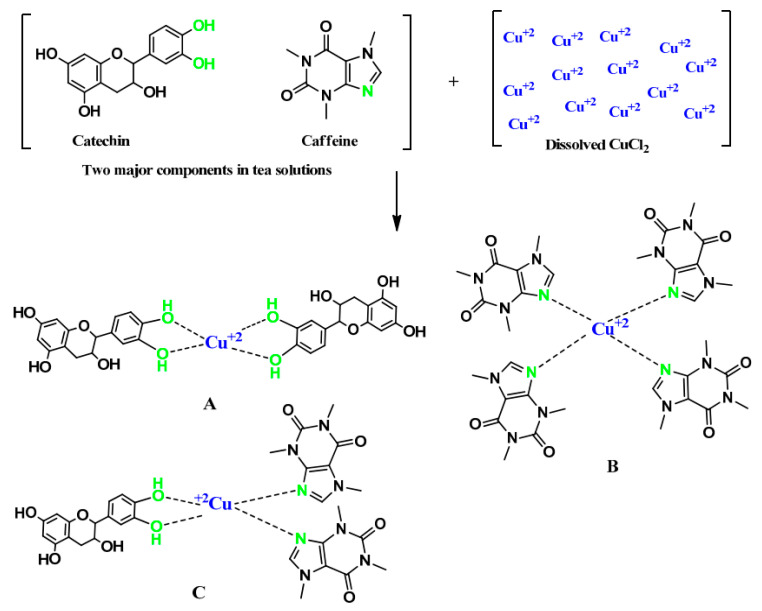
The proposed structure for the fabrication copper (II)-complex [118].

The most possible structure of the copper (II)-complex is shown in Figure 13. The copper (II)-complex is incorporated into PVA polymer in an attempt to produce a polymer composite with relatively low band gap energy. This composite shows a relatively high optical performance that is applicable in the photonic field. The effectiveness of the copper (II)-complex in reducing the band gap energy compared to the semiconductor copper nanoparticles and copper powder will be explained in detail in the band gap study section.

It is interesting to note that the colloidal copper complex absorption spectrum covers the complete optical range beginning from 1080 nm to 180 nm [118]. This kind of absorption spectrum is a characteristic of semiconductors and organometallic-based materials [119,120,121]. To recognize the formation of copper complexes rather than copper nanoparticles, there is no peak within the range of 600–800 nm for the former one [78].

Seku et al. [95] synthesized copper nanoparticles (CuNPs) by hydrothermal processes using a carboxymethyl gum kondagogu capping agent (CMGK). To evaluate the stability, UV–vis technique is informative. The synthesized CuNPs from copper chloride showed an absorption peak at around 558 nm. This appearance of the peak is considered strong evidence of NPs formation while it is absent in the copper (II)-complex absorption spectrum [122].

It has been documented that ultrathin Cu (II) modified BiOCl nanosheets (NSHs) is promising for photocatalytic and electrochemical energy storage applications [123]. The amendment of BiOCl NSHs with Cu (II) can substantially reduce the photocatalytic degradation activity and improve the electrochemical energy storage in symmetric supercapacitor (SC) [123]. It was reported that the performance of modified BiOCl NSHs with Cu (II) SC significantly improved. The 6 wt% Cu (II)/BiOCl showed a high specific capacitance of 155 F/g at 1 A/g with high specific capacitance retention of 101% after 5000 cycles, in addition to a very low charge transfer and solution resistance [123].

In another work chitosan-based membranes were modified with sodium hydroxide (NaOH) and glutaraldehyde (GA) solutions as covalent or ionic cross-linking agents, respectively [124]. The cross-linked membranes were used in EDLC cells as gel electrolytes, and the results have shown that the type of chitosan cross-linking agent may have an impact on the mechanical properties. Additionally, the fabricated EDLC cells exhibit the specific capacitance of 106 Fg^−1^ after the 10000th discharge cycle [124].

#### 3.2.3. Semiconductor Nanoparticles

Over the last decades, there is huge attention from researchers to fabricate semiconductor nanoparticles that are applicable in several technologies. As a consequence, miniaturization of semiconductor materials creates a breakthrough technologically. These small scales of materials modify both chemical and physical properties due to the large surface area and quantum size impact. The manipulation of conductivity, absorption and optical properties (refractive index and absorption coefficient) are increasingly necessary.

Herein, several applications are shown in different areas, for instance, packaging films, solar cells, nanoscale electronic devices, parts of automobiles, laser technology, light-emitting diodes, biosensors and chemical, waveguide, catalysts, components of armor, and superabsorbents. As far as we know, breakthroughs in nanotechnological fields are expected to be utilized in semiconductor industries. For example, in various electronic devices, different diodes including light-emitting diodes, analog and digital integrated circuits, and the silicon-controlled rectifier are extensively used. Moreover, several semiconductor nanomaterials are widely used at a large scale, such as AlGaAs, Si, CdS, HgCdTe, Si-Ge, GaN, GaAs, ZnSe, InP, InGaAs, SiC, AlGaN, ZnS, AlInGaP, CdSe, etc. The most popular applications are in computers, airbags, laptops, fiber networks, mobile terminals, TV remotes, cell phones, satellite dishes, traffic signals, car tail lights and palm pilots. The foremost semiconductors are II-VI and III-VI semiconductors that possess quantum confinement behavior in the radius range between 1 to 20 nm [125]. It is well-defined that miniaturization influences some of the physical characteristics (optical, dielectric, magnetic, thermal, structural, etc.) as a result of surface and quantum size impacts. More clearly, the very slight dimensions of these materials result in the alteration of physical properties that are fundamentally different from the bulk ones. Over a few years, considerable effort is devoted to exploring the impact of size and dimension reduction in semiconductors (on a nanometer scale).

This is ascribed to the large-scale usage in process and devices, such as catalysis, single electronic, optoelectronic, resonant tunneling, magnetic sensor, memory devices [126,127,128,129,130]. In the examination of the size evolution of the electronic structure, optical spectroscopy has been considered as an appropriate technique. NCs, especially semiconducting NPs have been utilized as fillers because of their unique luminescence and absorption characteristics.

The process of absorption of light in polymer NCs is different from that of metal NPs. It is not impossible to synthesize NPs with relatively small size from semiconductor elements that obey quantum confinement during the light absorption process. In these types of materials, there is an exciton phenomenon as a consequence of interaction with light, resulting in electron-hole pair formation. The radius of the exciton depends upon the band gap energy as well as the radius of the NPs. Likewise, the shape of the NPs determines the feature of the SPR peaks. Therefore, one can modify the band gap energy of semiconductor NPs via manipulating the size. This change in size (radius) not only reduces the band gap energy but also causes an alteration in the emitted photon wavelength. Now, the term quantum dots (QD) comes from the semiconductor NPs size and strongly correlated to the absorption wavelength [47]. NFs incorporation in polymers gives exceptional possibility to numerous optical uses. To comprehend the optical characteristics of such semiconductor-based NFs in polymer NCs, UV–vis spectroscopy is a negligible technique for this purpose. The NFs show emission and absorption features in the range of UV–vis on the radius of the particles, disparity, and band gaps in the polymer matrices [47].

It is facile and of great importance to use UV–vis absorption spectra in the analysis of quantum dot distribution, for example, CdSe or CdS incorporated into polymer matrices. Deeply, as the doped CdS diameter increases, a substantial shifting of absorption peak occurs, as reported by Wu et al. [131]. Moreover, an increase in dispersibility of CdS particles leads to absorption peak broadening whereas monodispersed CdS NPs as a result of tight attachment to the polymer cores result in sharper peak appearance. Tamborra et al. [132] examined the optical properties of CdS nanocrystals that are inserted in optically transparent polystyrene (PS) and PMMA. From the comparison of the absorption spectra of CdS NPs in both the solution of chloroform and in the PS and PMMA polymers, it was recognized that both polymers have not influenced the optical properties of native CdS nanocrystals. For example, there is a slight diminish in the polymer optical transparency at film state, the CdS optical absorption spectra are identical in solution and films. Consequently, based on these investigations both the PS and PMMA polymers behave just as stabilizers for the QDs [47].

**Figure 14:**
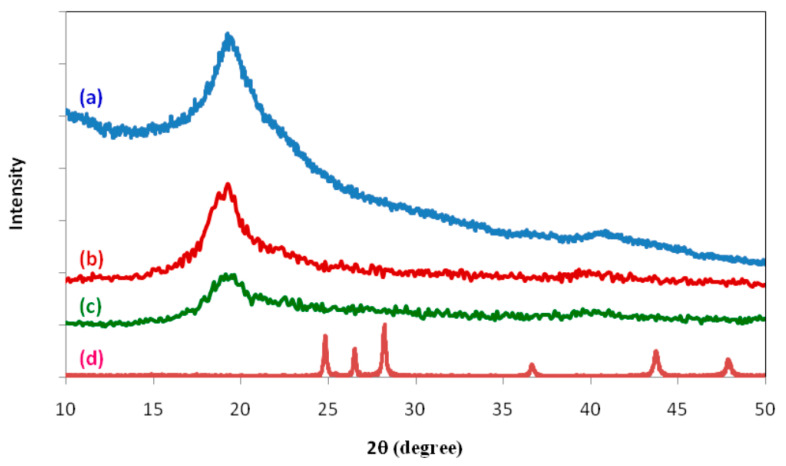
The XRD of: (**a**) pure PVA; (**b**) PVA incorporated 0.02 M CdS; (**c**) PVA incorporated 0.04 M CdS; (**d**) pure CdS [133].

Aziz et al. [133] synthesized composites of CdS nanoparticle/poly vinyl alcohol (PVA) with different molar concentrations ranging from 0.02 to 0.04 M of CdS. In the process of synthesis, chemical reduction was performed in situ followed by casting of the solution to produce films. To emphasize NCs formation, the X-ray diffraction (XRD) spectra were acquired for pure and PVA and CdS incorporated PVA NCs as shown in Figure 14. It was seen that a characteristic broad peak for pure PVA centered at 2θ ≈ 19.26° in the XRD pattern, indicating the semicrystalline of PVA [134,135]. This also evidences for relatively strong intramolecular hydrogen bonding within PVA monomer units and intermolecular hydrogen bonding between the monomer units [136].

Another interesting observation is the intensity attenuation of the peak of PVA in the NCs film, after embedding CdS NPs. This is attributed to the hydrogen bonding break between PVA chains hydroxyl groups and embedded CdS NPs, as a consequence, molecular chains are allowed to rotate to a large extent [137]. This finding of structural analysis using the XRD spectra of the PVA/CdS samples (Figure 14b,c) would not reflect the distinguishing peaks for CdS NPs (Figure 14d). One of the main reasons is the presence of a relatively very low CdS amount in comparison to that of the bulk PVA. Therefore, the domination of the amorphous nature contributes to the scattering and it was observed in the spectra of PVA/CdS samples [138]. Therefore, the possibility of exactly identifying the involvement of CdS in the whole scattering spectra is low [138].

**Figure 15:**
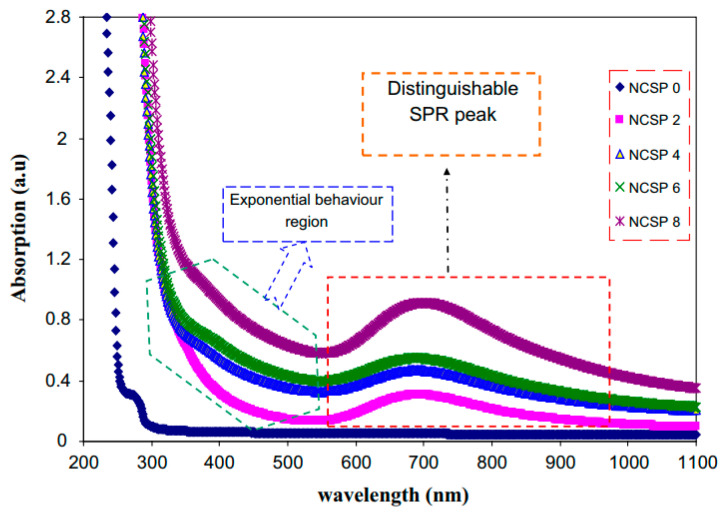
The pure PMMA and PMMA NCs absorption spectra of against wavelength. The films were specified as NCSP0, NCSP2, NCSP4, NCSP6 and NCSP8 for PMMA with 0 wt%, PMMA with 2 wt%, PMMA with 4 wt%, PMMA with 6 wt% and PMMA with 8 wt% of CuS, correspondingly [139].

Aziz et al. [139] synthesized CuS NPs in PMMA polymer successfully in situ at ambient temperature. It was noticed from the absorption spectra that an extensive absorption peak in the region between 570 and 980 nm wavelengths appeared. This has been identified as a typical SPR peak for CuS NPs. Figure 15 exhibits the pure PMMA and respective PMMA absorption spectra at 0 wt% to 8 wt% in Step 2 of CuS NPs. In the case of pure PMMA, there is a small absorption peak localizing in the UV region. It is also indicated that pure PMMA film has absorption which peaked around 280 nm that is typical of transitions of π − π* within the C=O [140]. Nevertheless, by increasing the doping percentage of CuS within the polymer matrices, a clear shifting in the absorbance has been observed around between 300 nm and 550 nm wavelengths. This is considered as an enhancement in absorption because of covering a wider absorption range of wavelengths. It was found that at 8 wt% of CuS, the sample showed higher absorption in comparison to less incorporated samples.

**Figure 16:**
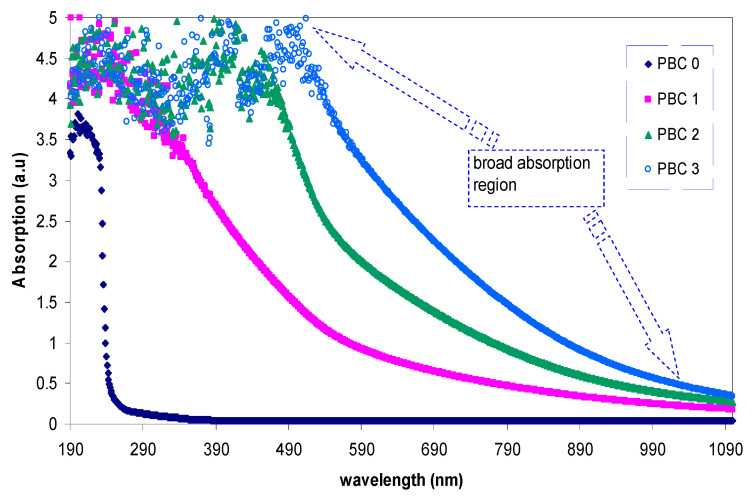
Optical absorption versus wavelength for pure PVA/PVP and PVA/PVP doped with various molar ratios of Ag_2_S semiconductor particles [144].

These indicate the capping of CuS NPs on the polymer chain through polar groups which participate in the π-electron delocalization. To pinpoint this finding, an increase in CuS causes an increase in absorption. It is also confirming a strong interaction between the PMMA polymer and CuS NPs [141]. There is a distinct peak between 550 and 980 nm as a characteristic of doped samples. This is related to the SPR phenomenon in which CuS NPs formed. In the literature, it was recorded that in the UV–vis spectra of NPs and their clusters possess a characteristic SPR peak [142]. It was also reported that the peak height reflects the concentration of the NPs [143]. Therefore, UV–vis spectra can be used to verify the NPs formation. The SPR phenomenon occurs only when a free electron exists giving a strong color to the films [142,143]. It worth noting that for longer wavelengths beyond 800 nm, a relatively tiny absorption was responded to. Furthermore, at a longer wavelength range, the dotted line absorption response can clearly be seen where constant for the whole NCs samples. Generally, there is a similarity in the whole change in the PMMA-CuS absorption spectra. This is mainly owing to this homogeneity in the structure of the samples [139]. In another study that was carried out by Aziz et al. [144], it has been revealed that Ag_2_S semiconductor particles impact the optical properties of PVA/Ag_2_S composites as shown in Figure 16. In the study, it was concluded that Ag_2_S semiconductor particles were not in the nanoscale in the absence of the SPR peak in the UV–vis spectra.

#### 3.2.4. Ceramic Fillers 

It is of significant importance to deal with polymer membranes in terms of relatively high performance, small size, safety and environmentally friendly. However, these polymer membranes are not free from drawbacks that cannot be produced on a large scale. Therefore, overcoming these drawbacks are priorities before use in large-scale applications. For example, commercially available polymer electrolyte membrane (e.g., Nafion) has been limited due to the high cost, which consequently influences the entire cost of the devices. This polymer membrane also suffers from conductivity reduction at high temperatures, especially beyond 80 °C, which in turn reduces the fuel cell performance [145]. To overcome these limitations, a number of research groups have devoted the efforts in composite membranes. An improvement in the optical and electrochemical characteristics of polymer electrolytes are carried out by adding ceramic fillers as will be discussed later.

Ceramic materials have a characteristically brittle appearance with small dielectric strength and it is difficult to be manufactured because of the necessity for high temperatures in the production process. On the other hand, polymers are characterized by flexibility and ease of processing at low temperatures and stable under large dielectric breakdown fields [146].

It is worth noting that many polymers are eligible as matrices that interact with additives in an attempt to form composite materials. This is owing to strong adhesive behavior, in addition to relatively high resistivity toward a corrosive environment, light weight and versatile mechanical performance. The polymer materials are insulators that have small dielectric constant and large dielectric strength as well [147]. The first incorporation of ceramic filler (γ-LiAlO_2_) into a polymer electrolyte was performed by Panero et al. in (1992) [70]. This was in an attempt to increase the mechanical strength of polymer electrolytes of PEO–LiClO_4_. Deeply, the incorporation of ceramic fillers into polymer electrolytes strengthens the characteristics of interfacial due to the decrease in the interface resistance [148,149]. Additionally, ceramic fillers can reduce the degree of crystallinity and promote optical properties as well. Generally, the ceramic fillers are divided into two primary types: active and passive ceramics. The active ones contribute to the mechanism of conduction e.g., Li_2_N, LiAl_2_O_3_; while passive fillers, for instance, Al_2_O_3_, SiO_2_ and MgO remain out of the lithium transport process. 

A huge number of nanomaterials have been used in preparing organic/inorganic NCs. For example, TiO_2_ NPs as inorganic fillers have a wide usage. It possesses satisfactory stability, high refractive index and high hydrophilicity. In addition, TiO_2_ NPs have absorption in the UV range, harmlessness and good transparency for optical light. Moreover, it is defined as a matter with a robust trend for redox processes and also for photoelectrochemical cells [150]. Furthermore, air purification and water [151], degradation of the organic contaminants are the most popular applications of these kinds of nanomaterials because of the great activity of photocatalytic and inexpensive availability of TiO_2_ nanomaterials [152]. The presence of TiO_2_ NPs like pigment in the form of powder provides whiteness to goods, such as coating plastics, food packaging material, paints, papers and ink [153]. In all these uses, manipulation of particle radius and well dispersion of TiO_2_ in the polymer matrices are critical factors [154].

The addition of inorganic particles into polymer matrices to form composite materials means the production of new ones that are completely different from conventional materials. For example, TiO_2_ NPs exhibit a strong tendency to aggregate that can be directly added into the organic coating. This is due to the high surface area and high polarity of TiO_2_nanoparticles. However, the challenge that faces this modification using TiO_2_ is the uniform dispersion of TiO_2_ NPs. Nevertheless, it is solved via using ultrasonic irradiation [154]. Many efforts have focused on examining the impacts of ceramics within the polymer matrices on mechanical and optical properties [155,156]. It is known that the optical properties of polymer/Al_2_O_3_ NCs, for instance, photonic and light emission characteristics, rely on the shape and size of the NPs, concentration and spatial distribution within the rest of polymer matrices [157].

The presence of NPs attenuates the transmittance of the light as reported for polyimide-Al_2_O_3_ [158]. Clearly, the addition of 2% of Al_2_O_3_ attenuates 70% transmittance at 600 nm and as the amount is increased, the attenuation increases as well. This can be attributed to the aggregation of NPs within the composites causing significant light scattering [155].

Hakimelahi et al. [159] used in situ polymerization blending technique in examining the efficiency of alumina nanowhiskers (NWs) dispersion within polycarbonate (PC) composites. The results showed that at 0.5 and 1 wt% Al_2_O_3_ NWs for the base PC and NCs, the films were clear as in the case of the pure PC. In contrast, at 2 wt% of loading, haziness, unclear and misty were observed at various temperatures. This is in agreement with the great aggregation formation and/or existence of voids where light diffracts. Interestingly, in the sample of PC–Al_2_O_3_ composite, there is transmittance against wavelength, showing absorption of ultraviolet and also significantly increases with rising alumina [155].

Chandra et al. [160] studied the effect of the addition of NPs into the PC/Al_2_O_3_ (NCs) via measuring the light transmittance percentage in which the percentage decreased as the amount of alumina was increased. It was also recorded that lower light transmittance was counted for the poly (styrene-maleic anhydride) copolymer-coated alumina/PC NC compared to the untreated composite with alumina. Ritzhaupt-Kleissl et al. [161] confirmed the optical property changes of polymers upon the addition of alumina. Reforming PMMA resin with silane-modified Al_2_O_3_NPs can be seen from this change in the refractive index. It was also recorded that there is an improvement in optical transmittance in the IR and visible regions compared to that remain untreated.

**Figure 17:**
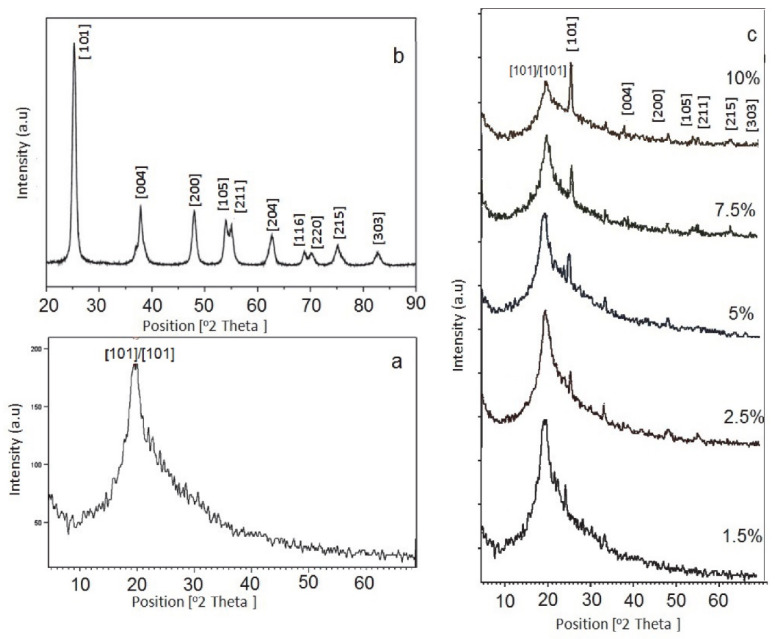
X-ray diffraction pattern for (**a**) pure PVA, (**b**) pure TiO_2_ and (**c**) TiO_2_/PVA composites [154].

Shehap and Dana [154] reported polymeric composite film preparation using polyvinyl alcohol (PVA) doped with titanium dioxide NPs at different proportions (1.25, 2.5, 5, 7.5, 10% TiO_2_/PVA) through casting techniques accompanying sonication. It was shown that with increasing TiO_2_ content into PVA polymer matrix, the amorphous domain increased as evidenced by the XRD test. In order to investigate the degree of crystallinity of pure and TiO_2_/PVA polymer NCs, X-ray diffraction has been conducted. The XRD of pure PVA, pure anatase TiO_2_nanoparticle and the composites (1.25, 2.5, 5, 7.5 and 10 wt/wt/PVA) in the 2θ range between 5° and 70° are shown in Figure 17. A broad peak lying between 2θ equates to 15° up to 30° was noted for the XRD pattern of pure PVA, which involves both the crystalline and amorphous regions [162]. For the pure TiO_2_ anatase the spectrum is in accordance with that the fingerprint documented in the literature [163]. However, in the doped films with 1.25% and 2.5% TiO_2_ /PVA, the peak of PVA (2θ = 19.8°) is observed in the samples along with only one peak of TiO_2_. 

Importantly, an obvious peak centers at 2θ=25.3° and a new one at 2θ = 32° that are not seen for individual components (pure PVA and TiO_2_) indicate the stirring effect by ultrasonic waves that produced dissociated NPs. It is also observed that there is an interaction between PVA and TiO_2_. An interesting observation is that other percentages (5, 7.5 and 10 wt% of TiO_2_) that doped into PVA were not effective in disappearing any indexed peak of the NPs. The semicrystallinity of PVA has been changed to the amorphous phase as a consequence of the addition of TiO_2_ as evidenced by the XRD. It is seen from the XRD that broadening width and decreasing intensity of the peaks are caused by doping. It is chemically understood that disruption of intramolecular interaction via H-bonding between the chains of macromolecules of PVA results from the addition of TiO_2_. Consequently, two factors govern the degree of lowering crystallinity; the quantity of loaded TiO_2_ and the extent of dispersion in the composite samples. The chemistry of the addition of TiO_2_ into PVA can be explained on the basis of the possibility of an association between Ti^+^ ions and hydroxyl groups within the PVA. This is evidenced by the appearance of a peak at 2θ = 32° [154].

**Figure 18:**
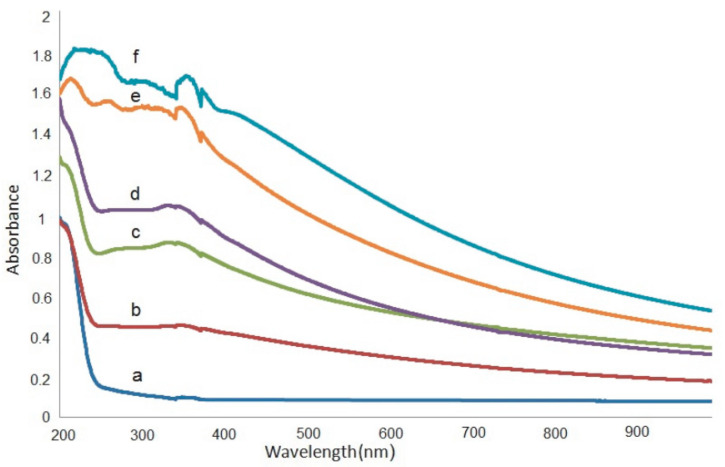
UV–visible absorption spectra for (**a**) pure PVA, (**b**) 1.5, (**c**) 2.5, (**d**) 5, (**e**) 7.5 (**f**) and 10 wt% TiO_2_ [154].

The UV–vis spectra of pure PVA and doped PVA composites are presented in Figure 18a–f [154]. In an explanation, pure PVA has shown a band at 198 nm wavelength which is attributed to the transition of π − π⃰, as well as the absorption band at 281 nm, is correlated to n − π⃰. Furthermore, another shoulder of a peak is observed roughly at 208 nm, indicating the existence of unsaturated bonds of carbonyl (C=O) and/or alkene (C=C) primarily in the pure PVA tail-head [164]. The characteristic absorption of TiO_2_ appears in the form of a broad absorption band in the TiO_2_/PVA composites, and the intensity relies upon the quantity of TiO_2_ load in the composites. It is also seen that the absorption peaks for the PVA appeared in all the composites. It is interesting to notice that the absorption rises generally with increasing the dopant quantity. All these emphasize the existence of strong interaction between the NPs of TiO_2_ and PVA, suggesting new material formation.

## 4. Carbon-Based Nanomaterials

### 4.1. Carbon Nanotubes

Carbon nanotubes (CNTs) are described by Sumio Iijima in 1991 that consist of thin carbon fibers at the nanosize scale and microsize in length. These types of carbon materials have been widely utilized [165]. The structure of the CNT is composed of an enrolled graphite sheet in a planar-hexagonal arrangement of carbon atoms in honeycomb lattice appearance [165,166]. The nanotubes (NTs) are classified into multiwalled carbon NTs (MWCNTs) and single-walled carbon NTs (SWCNTs) where each type forms depending on the preparation method [165,167]. The MWCNTs are central hollow cores that are composed of two or more concentrated cylindrical shells of graphene sheets coaxially arranged. The SWCNTs are single graphene layers that were rolled up into a seamless cylinder [168,169].

A relatively high electrical and conductive properties and mechanical strength of CNTs, encourage researchers to investigate increasingly. In addition, CNTs with no defects possess an elastic modulus of approximately 1 TPa and a relatively large tensile strength of about 300 GPa [166,170]. In fact, the existence of a strong covalent bond between the carbon atoms and the characteristic geometric arrangement in cylindrical shapes give these exceptional properties [171]. These properties of carbon NTs have drawn great attention to research groups to be used, especially in polymer NCs synthesis [172]. The utilization of carbon NTs in polymers has been extensively conducted in an attempt to form composite materials with unique mechanical, optical, electrical and thermal properties. However, the difficulty of dispersion of these materials is a challenging task. Herein, there is a strong tendency of CNTs to aggregate throughout dispersion courses because of no association between CNTs and polymers. Nevertheless, the chemical conditions will change the extent of CNTs dispersion within polymer matrices. To increase the interaction between CNTs and polymers, surface functionalization has been performed chemically [173]. The insertion of functional groups on the surface of CNTs walls increases the chemical reactivity toward the fillers. This induces a better interfacial region, consequently a relatively much loading into the matrices [171]. Herein, a modification of the carbon surface of NTs is explained using a combination of nitric as well as sulfuric acids (HNO_3_/H_2_SO_4_) where carboxylic acid groups (–COOH) can be introduced [171]. This modification has been established by Goyanes et al. [174] and through this, the CNTs surface becomes more compatible with the polymer matrices.

It is obviously seen throughout the literature that CNTs and graphene (Gr) have been utilized extensively. Those materials create a breakthrough in electronics and energy fields. However, several issues have to be taken into consideration, such as low solubility leads to the difficulty of processability in common solvents which limit the utilization of these materials at a large scale. To overcome these difficulties; numerous techniques were used for CNTs and Gr dispersion into solvents as well as polymers. For example, choosing appropriate matrices for the carbon-based nanomaterials dispersion is an option. It is also helpful to involve binds to the surface of the carbon framework via noncovalent interactions. Thus, these approaches solve the aggregation of the components in the composites, in other words, enhancing material dispersion. Furthermore, the π–π reaction among the carbon framework of CNTs and Gr on one side and of the conducting polymers on the other side can be exploited using conjugated block copolymers, for instance, poly(3-hexylthiophene) (P3HT) block copolymers [47,175].

Romero et al. worked in 1996 in an effort to synthesize a nanotube/polymer photovoltaic device [176]. The study involved an MWNT layer that was deposited on Teflon, and then a PPV derivative was casted with a thin layer of aluminum at the top. The characteristic of the device was the perfect behavior of diodes with just a tiny photocurrent moved after being irradiated with the He–Ne laser light. This may have been owing to the thick active layer of PPV. In another work carried out by Ago et al., the fabrication of NTs included the device of photovoltaic by depositing a PPV layer over a glass-supported MWNT film [177].

To test the extent of interaction between MWNTs and PPV, it is of the best choice to use photoluminescence (PL) measurements. It has been noticed that the PL efficiency of the PPV is determined to be significantly decreased by the MWNT layer existence. In response to radiative recombination, photoluminescence results decreased, which might imply an improvement in exciton separation that is desired in a photovoltaic device [178]. CNTs and carbon-based nanomaterials were broadly studied. In recent publications, a novel class of carbon-based material has been utilized which is called carbon quantum dots (CQDs). This new carbon material has shown a potential alternative to CNTs as a sustainable material for large-scale applications [179]. The carbon nanodots (CNDs) have been extensively studied and appeared as one of the interesting carbon materials in the mid-2000s [180]. The CNDs are NPs of less than around 10 nm in size that normally consist of carbon, hydrogen, oxygen and nitrogen. The most utilization of these carbon materials is in analytical chemistry because of brilliant fluorescence. Furthermore, these materials can be tuned in visible regions. This carbon-based material is superior in some aspects, for instance, biocompatibility, ease of manipulation, good solubility in water, harmless and inexpensive. In addition, the large sensitivity to perturbation in the outside environment and noticeable ability for electron accepting and donating are two impressive characteristics of this material. Therefore, the previous two behaviors make this material to be used in CDs in a great extensive range of uses, such as optoelectronics and sensors. Practically, the CDs optical characteristics are comparable to a large extent and even competitive to fluorescent semiconductors [181].

To be applicable, the surface of CNDs is modified by insertion of oxygen/hydrogen-containing species as fillers to be able to form H-bonding, for example, –OH and –COOH [182]. In these materials that involve C-based equivalents of optically active quantum dots (QDs). There is no existence of poisonous elements, normally heavy metal atoms which limit, especially, the QDs usage in the application of bio-oriented. To be widely applicable in optoelectronic devices, two properties are taken into consideration, which are the CDs bright emission and the noticeable electron-donor [183,184]. The sensitivity of the fluorescence technique of these material-based metal ions in solution is utilized to produce nanosensors [185]. Furthermore, these materials to be used in drug delivery experiments which is the key property is nontoxicity [186]. Moreover, CDs are extensively applicable, for instance, antiviral [187] and antibacterial [188]. In addition, CDs have been recently used as nanoweapons against mosquitoes [189] and as one of the components of fluorescent inks [190]. However, the mainstream of CDs-based devices was still improved as well as checked in a laboratory scale. Despite small-scale applications, commercialization is possible in the near future.

**Figure 19:**
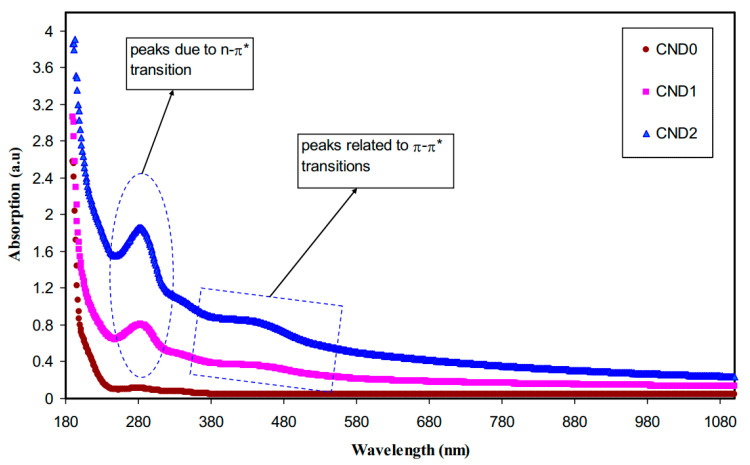
Films absorption spectra. Obviously, with a rising amount of carbon nanodots (CNDs), the absorption changes to longer wavelengths. All films were specified as CND0, CND1 and CND2 equivalent to doped PVA solution with 0 mL, 15 mL and 30 mL of 5 mg of dissolved CNDs, correspondingly [191].

Aziz et al. [191] synthesized CNDs from glucose via using the hydrothermal treatment and solution casting methodology and also added into PVA polymer to prepare PVA/CNDs polymer composite films. Figure 19 shows the absorption spectra of pure PVA and the doped ones for various ratios using CNDs: 0 mL, 15 mL and 30 mL. It is clearly seen that two distinct peaks appear in the UV range. From the spectra, evidence of modified PVA is obtained. It is well-known that the light absorption in the area of UV–vis causes electron transition in σ and π and n-orbitals from the state of ground to the larger excited state based on the theory of molecular orbital [192]. More specifically, the two detected peaks centered at 280 nm and 430 nm are assigned to the transitions of n − π* and π − π*, correspondingly [193]. It is noted that the PVA/CND composites absorption extended from 580 nm to UV region. The more interesting observation is that as the CNDs amount rises, the intensity of the peaks due to the transition of n − π* as well as π − π* also rises. This might be correlated to the –OH and –NH_2_ groups’ distribution on the surface of the CNDs [182].

**Figure 20:**
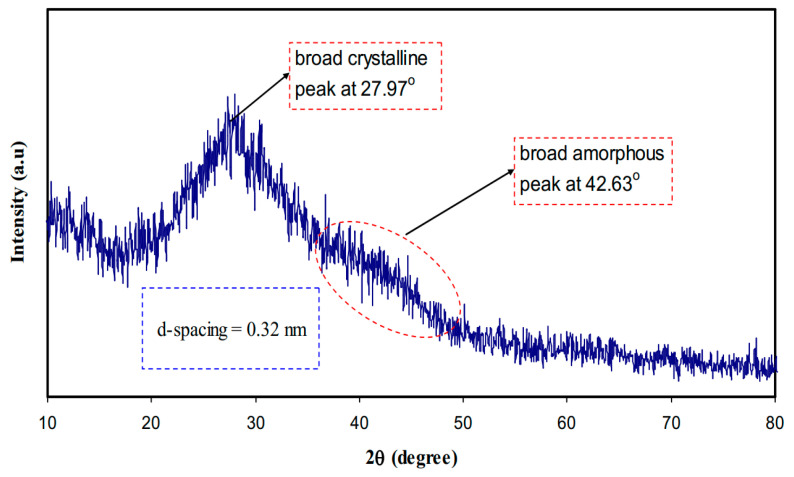
CN-dots XRD spectrum at room temperature [191].

From the XRD analysis, it was obtained that an obvious sharp peak at 2θ = 27.97° (crystalline peak) appears whereas a broad peak (an amorphous peak) lies at 42.63° as shown in Figure 20. It has been explained that the sharp and broad peaks reflect crystalline phase (ordering structure) and amorphous phase (disordering structure), respectively [194]. An interesting observation in the spectra is the appearance of a peak at 2θ = 20° in pure PVA which can be attributed to the existence of semicrystalline structure [66]. Figure 20 exhibits the XRD pattern recorded for the C-dots. The feature of the pattern in the current work is entirely dissimilar where it relatively borders that of the former one [195]. Importantly, the d-spacing values of the C-dots obtained in this study and that in the literature are different where 0.32 and 0.34, respectively [195,196]. It is satisfactory evidence to see the XRD extensive peaks for prepared nanoscale particles [139,197]. The crystalline size from the Debye–Scherrer formula was estimated for the greatest peak in the XRD spectrum at 27.97° is 1 nm for carbon nanodot (CND) particles as shown in Figure 20. At the same time, the broad XRD peak provides the estimated C-dots particles tiny radius. It is worth mentioning that a carbogenic core composed of amorphous and crystalline phases is the characteristic feature of C-dots that are enriched in the functionalized surface. One can note that in the case of C-dots, the amorphous part is the dominating part [191,195].

**Figure 21:**
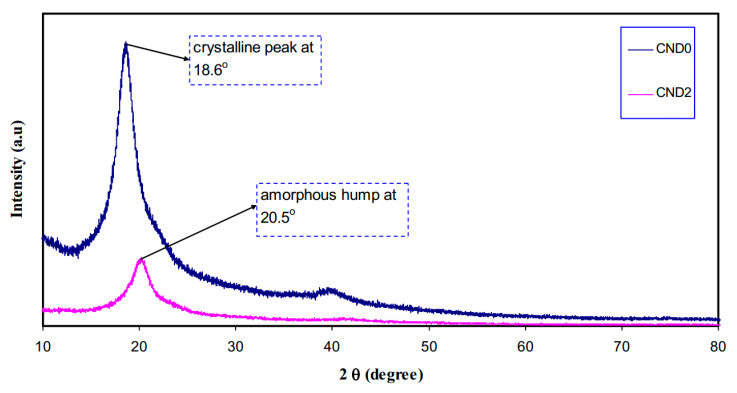
Pure PVA and PVA/CN-Dot XRD spectra. It is vital to observe that the PVA main peak is extra widened and its intensity reduced after the insertion of CN-dots. All films were specified as CND0 and CND2 equivalent to doped PVA with 0 mL and 30 mL of 5 mg of dissolved CNDs, correspondingly [191].

The authors used the XRD pattern for pure and doped (CND2) PVA films (Figure 21) as an evidence of interaction among PVA and CNDs particles. For example, the shifting of the significant peak from 18.6° in pure PVA to 20.5° in doped PVA is reasonable evidence. Moreover, the other two expansive peaks appear at 2θ = 23.4° and 41.18°. Clearly, the three peaks centered at 2θ = 41.15°, 23.43° and 20° correspond to (111), (200) and (101) crystalline phases of PVA, respectively [198]. Importantly, the peak shifting at all positions proves the occurrence of enough interaction between the functional groups in PVA and CNDs via –OH in PVA and the surface of the NPs of CND. This causes disruption within PVA followed by formation of amorphous phase in the whole composites [199]. The estimated crystalline size from the Debye–Scherrer equation was carried out for 18.6° as well as 20.5° which were 2θ = 6.5 and 4.6 nm in PVA (CDN0) and incorporated PVA (CND2), respectively. As a consequence, addition of CNDs particles into PVA lowers the crystallite size of regular phases or PVA chains in the region of crystalline. Therefore, amorphous domination in PVA doped composite (CND2) is evidenced strongly from reducing in intensity and extending of the peaks. The peak’s absence in the case of CNDs in incorporated PVA demonstrates the dissolution of the entire CNDs in the body [191].

### 4.2. Graphene

Carbon as a fascinating element lies in the top of the 6th column in the periodic tables has always remained interesting from the perspective of scientists. Herein, a number of carbon allotropes are mentioned, such as fullerenes, diamond, carbon NTs, graphite and recently described Gr. The breakthrough of the carbon has appeared since the discovery of fullerene and Gr and two Nobel Prizes awarded in the years 1996 and 2010 to Curl, Kroto and Smalley and Geim and Novalec, correspondingly. There are a huge number of published works on graphite intercalated [200], fullerenes (1985) [201] and carbon NTs (1991) [202]. Since 2004, many publications on graphene have been recorded [203,204].

**Figure 22:**
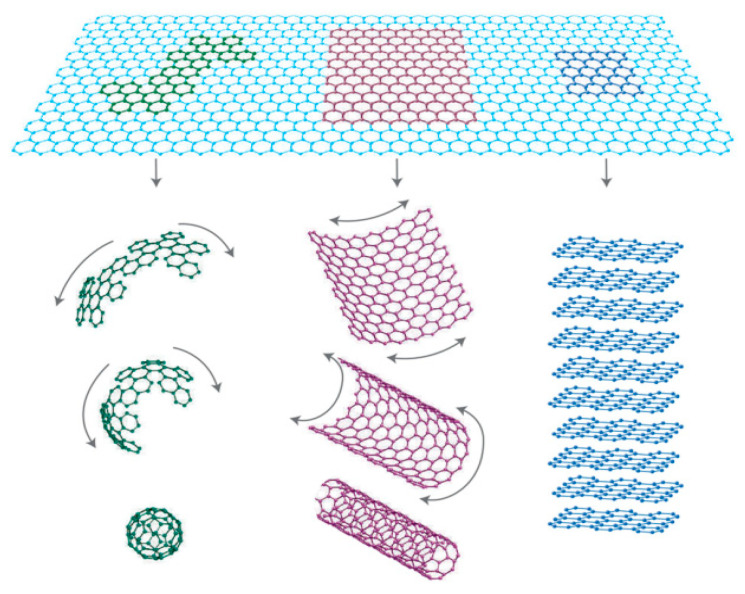
Graphene is a 2D building block of graphitic forms. It wraps to create 0D buckyball, rolls to create 1D nanotube, as well as stacks to create 3D graphite [206].

The Gr is a two-dimensional (2D) honeycomb lattice of a flat carbon atoms monolayer where closely packed. The carbon atom is completely conjugated sp^2^ hybridized that is structurally planar and considered as a fundamental building block for graphitic materials of all other dimensionalities (see Figure 22). It can wrap up into 0D fullerenes, roll into 1D NTs and stack into 3D graphite [205].

Nowadays, Gr has been extensively used in synthesizing various new inorganic and organic materials. Therefore, the Gr-based composites have been enormously utilized in energy production devices, for example, batteries [207], SCs [208], fuel cells [209] and sensing platforms [210]. To be more applicable, the Gr has to be multifunctionalized in polymer to create developed multifunctional composites as one of the likely routes [211]. Involvement of polymer materials in route typically possesses special specific modulus and strength in which they have extensive applications in aerospace, defense and automobile industries.

The appearance of nanotechnology encourages researchers to use many NFs in polymers, for instance, nanosilica, carbon black and CNTs, so as to enhance mechanical, optical, gas barrier, thermal and electrical characteristics. The superior of Gr over CNTs can be seen from the larger ratio of surface to volume and inaccessibility of the inner NTs surface to polymer molecules [212]. In recent years, Gr and its derivatives were doped into an extensive range of polymers, for example, polypropylene (PP) [213], nylon [214], epoxy [215], polystyrene (PS) [216], polyaniline (PANI) [217], poly methyl methacrylate (PMMA) [218] and polyethylene terephthalate [219] for numerous uses.

However, the fabrication of Gr-polymer NCs faces several problems. These factors include the kind of Gr and its derived, their intrinsic characteristics, such as the exfoliation and dispersion of Gr in the polymer matrices, the interfacial reaction among the graphene and the matrices and the natural network structures of Gr. In other words, the compatibility between the Gr and polymer leads to the production of new materials (composites) with useful properties [206]. Gr and its derivatives also show additional interesting properties, for example photonic characteristics owing to unique composition (one-atom-thick layer). This enables Gr and its derivatives to be transparent with insignificant reflectance below 0.1% to optical light, and also strong interaction with photons resulting from Van Hove singularity [220,221].

**Figure 23:**
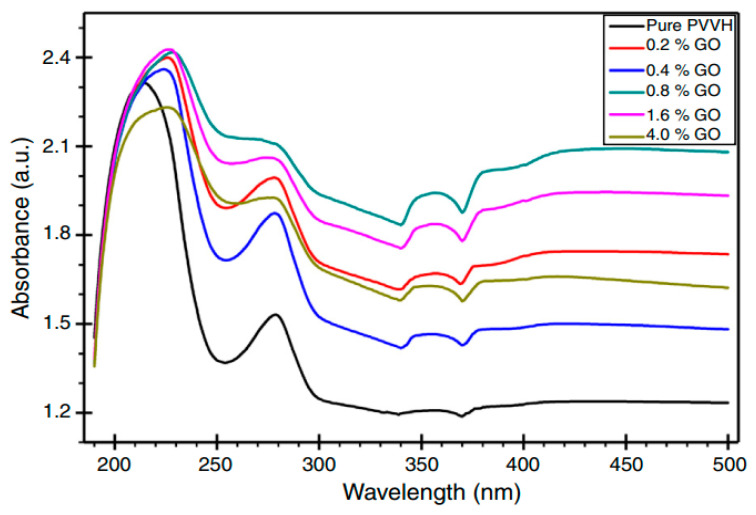
Pure PVVH and PVVH/GO nanocomposites absorption spectra in the range from 190 to 500 nm at ambient temperature [229].

Contrary, the band gap of zero energy results from complete overlapping between the valence and the conduction bands; therefore, limits utilization in both photodetectors and photovoltaics. Therefore, the band gap energies of materials have to be changed via structure reforming [222], electric field [220] and doping with suitable chemical compounds at the expense of other properties [223]. Niyogi et al. [224] developed an approach in an attempt to alter the band gap of graphene by covalent functionalization. This development has been carried out by attaching groups of nitrophenyl straight on the sheets of Gr where spontaneous electrons migrate from graphene top-nitrobenzenediazonium tetrafluoroborate facilitating considerably. The change in the band gap is based on the principle of converting the hybridization of carbon atoms from sp^2^ to sp^3^, giving a ~0.4 eV band gap energy. This manipulation of band gap is resulted from surface functionalization of Gr, producing useful semiconducting nanomaterials [224,225]. Fang et al. [226] studied an effective methodology to functionalize Gr by introducing hydroxylated aryl groups to the surface of Gr by covalent bonding. Alternatively, diazonium addition reaction has been performed where working like initiators for atom migrate radical polymerization of polystyrene. In fact, it is of great importance to fabricate a NCs from Gr and polystyrene, which in turn significant improvement of mechanical properties occurs [226]. One may ask why Gr and its derivatives including Gr oxide have wide applications? The answer is because of the unique properties of these materials, for instance, a relatively high mechanical stability, and good magnetic, electrical, optical, thermal characteristics [227]. The Gr oxide is provided from inexpensive natural graphite industrially. The surface of Gr can be enriched with several functional groups, such as carbonyl group, carboxyl group, hydroxyl group and epoxide. The functionalized surface of Gr with oxides can significantly enhance the interfacial reaction with polymer matrices [228]. Yassin et al. [229] synthesized Gr oxide developed by Hammer’s technique [230] from nanopowder of graphite oxide with an oxidation degree in the range of 5–10%. In implementing this methodology, various proportions: 0.2, 0.4, 0.8, 1.6, 3 and 4 wt% of Gr oxide (GO) have been effectively inserted into poly (vinyl chloride-co-vinyl acetate-co-2-hydroxypropyl acrylate) (PVVH) copolymer. The authors showed the absorption spectra in the range of 190–500 nm of pristine PVVH and PVVH/GO NCs at room temperature as presented in Figure 23.

The most important observation in these spectra is the appearance of a characteristic absorption sharp edge at 234 nm. The response of the loaded polymer with Gr oxide reflects in the appearance of a broad absorption edge. This could be due to the occurrence of disordering in the phase of the composite matrix. This means the amorphous phase dominates the matrix as evidenced by XRD patterns in Figure 23 [231]. From the optical study, an absorption band centers at about 214 nm noted and caused by the n→π* transition; whereas, the π→π* transition of electrons in carbonyl groups (C=O) and/or unsaturated (C=C) within the composites of the polymer and Gr oxide is responsible for the appearance of another band at 280 nm [232,233]. The latter band appears as a shoulder-like peak with increasing the filler quantity and undergoing shifting toward longer wavelength, confirming a strong association between the filler on one side and the PVVH copolymer.

**Figure 24:**
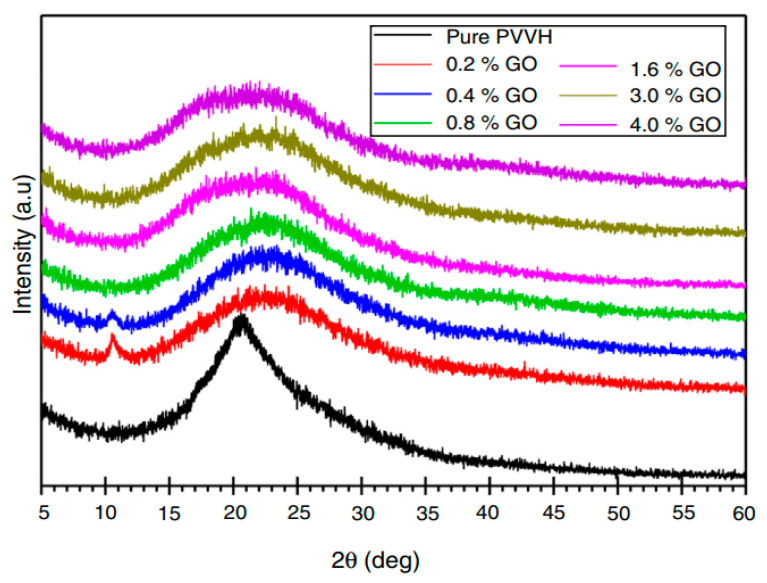
XRD spectra for the pure PVVH copolymer and PVVH including dissimilar GO levels [229].

The authors also conducted the XRD spectra of pure PVVH copolymer and PVVH occupied with changing levels of Gr oxides indicated in Figure 24. The primary peak concentrated at nearly 21.8° is attributed to the pure PVVH. The wideness of this peak was raised with intensity decline up on dispersing graphene oxide (GO) in the polymer.

This is in a good agreement with that concluded by Hodge et al. [234], emphasizing dominancy of the amorphous phase in the composites. In the structural analysis using XRD, a peak at 10.5° is attributed to GO corresponds to (0 0 1) plane [235]. An interesting observation is the appearance of a peak for GO even at relatively low concentration (0.2 and 0.4 wt%) and absence in the rest of the samples, indicating a complete insertion of GO into the host matrices. This means that the distinguishing peak of GO in the composite XRD pattern decreases gradually in intensity with raising the amount and entirely disappears at high amounts. Moreover, it provides strong evidence that GO sheets have been effectively exfoliated into single sheets and well dispersed within PVVH at molecular levels [229].

## 5. Optical Parameters

### 5.1. Refractive Index Study

Refractive index is one of the fundamental physical quantities of materials that represents electromagnetic wave propagation through a given medium that is mathematically expressed as follow [236]:

(1) $$n=\;\frac{c}{v}$$

where *υ* is the light velocity in a medium, *c* is the light speed in space. If a material absorbs the incident light, the refractive index equation can be modified using the following equation [236]:

(2) N=n+iK

where *N* stands for the complex refractive index, *n* stands for the real refractive index, *K* stands for the extinction coefficient which relates to to the absorbed light [236].

It is well-known that polymers in general have wide applications, such as plastics, coatings and transparent materials. Therefore, determination and analysis of the optical properties of polymers are of significant importance. The transparency property is in association with the nature of the crystallinity of polymers [118]. Although most of the polymers are colorless and transparent, some others are colored and opaque, for instance, poly acetylenes and phenolic resins. The transparent ones allow visible light to pass and the opaque polymers can be obtained using colorants, flame retardants, fillers, gases, stabilizers and moisture [118,237]. The refractive index, n as an optical property reflects the ability of polymers to bend and reflect light. For the magnitude of the refractive index, n relies on the identity (chemical composition) of materials, for example, 1.33 and 1.0 are recorded for water and air, respectively [118]. Moreover, the refractive index, n of most polymer materials and titanium oxide pigment are 1.5 and 2.5, respectively. The value of the refractive index of 1.3 to 1.7 for conventional polymers has been documented [238] while higher value has been reported for most inorganic materials [239,240]. Interestingly, the crystalline materials have exhibited a relatively high refractive index that is independent of both light strikes on the surface and wavelength. It is also proved that the light velocity migrating within polymers is impacted by the polarity of the primary bond within the molecules. The polarizability *P* is directly proportional to the molecular weight per volume (*M*) and inversely to the density (*ρ*) as shown below (the Lorenz-Lorenz relation) [237]:

(3) $$P=\left(\frac{n^{2}-1}{n^{2}+1}\right)\frac{M}{\rho }$$

Another optical important property is polarizability, *P* that in polymer materials is related to the number of molecules per unit volume and identity of the molecule. In other words, each molecule has its characteristic electron system in which the number and mobility of electrons determine the polarizability. For example, the polarizability of hydrogen is much smaller than carbon, and neglecting the effect of the former is taken in the calculations of whole organic polymer polarizability. 

To quantify the illumination fraction transmitted through a material, Beer–Lambert established the following relationship [118]:

(4) $$\frac{I_{T}}{I_{0}}=\;e^{-\;\alpha d}$$

where the fraction of light incident and transmitted are represented as *I_o_* and *I_T_*, respectively. The ratio of incident to transmitted light is dependent upon the light path length, *d*, and the polymer materials absorptivity coefficient at specified light wavelength. One outstanding issue related to this relationship when the light passes through a homogeneous material for example a perfect amorphous polymer or a crystalline ordered polymer, is the occurrence of interference.

In heterogeneous materials, the passed light undergoes variation in polarization as a result of different phases (crystalline and amorphous). When light passes through air into a solid medium, it undergoes several phenomena, such as reflection, absorption and transmission. Therefore, the total light intensity of the incident one (*I_o_*) at the second surface equals the summation of reflected, absorbed and transmitted light intensities as designated as *I_T_*, *I_R_* and *I_A_*, respectively [118]. The following simple algebraic equation shows the summation of all intensities [118]:

(5) $$I_{0}=\;I_{R}+\;I_{A}+\;I_{T}$$

The measured radiation intensity in watts per square meter denotes the energy migrated per unit time per unit area which is perpendicular to the direction of radiation propagation. Therefore, by dividing Equation (5) by *I_o_*, following relation can be obtained [118]:

(6) R+A+T=1

where *T*, *R* and *A* stand for the transmissivity (*I_T_*/*I_o_*), reflectivity (*I_R_*/*I_o_*) and absorptivity (*I_A_*/*I_o_*), correspondingly, or the fractions of incident light are transmitted, reflected and absorbed. In other words, the whole incident light that undergoes reflection, absorption or transmission, must equal unity [49,118].

(7) $$\frac{I_{T}}{I_{0}}=T$$

(8) $$T=\;10^{-A}=\;e^{-\;\alpha d}$$

In order to fabricate a material that is optically active, the refractive index has to be modified which makes it efficient optically, for example, metal complex-based materials possess relatively high optical activity [241]. A deep and comprehensive understanding of the refractive index is vital for designing optoelectronic devices. Theoretically, several factors govern the refractive index, such as polarizability and density of the medium, in addition to the temperature and pressure [242]. To calculate the refractive index (n) of a film, the reflectance, absorbance and coefficient of absorptivity (extinction coefficient) have to be known as shown below [118]:

(9) $$n^{*}\left(\lambda \right)=n\left(\lambda \right)+K\left(\lambda \right)$$

where the extinction coefficient is symbolized by *K* and the refractive index is labeled by *n*. The relation between the *K* and *n* values is indicated as follows [118,243]:

(10) $$n=\left(\frac{1+R}{1-R}\right)+\;\sqrt{\frac{4*R}{{\left(1-R\right)}^{2}}}-\;K^{2}$$

where, *K*($\frac{\alpha \lambda }{4\pi t}$) is the extinction coefficient in Equation (10), which is directly proportional to the wavelength (*λ*) and absorption coefficient (*α*) while inversely proportional to the thickness (*t*) of the films [85,118].

**Figure 25:**
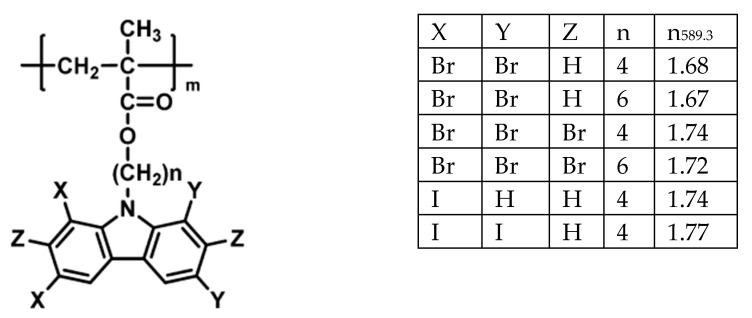
Halogen substituted polymethacrylates structure and values of refractive index [250,251].

Therefore, to have high performance eyeglass lenses and optical fibers, it is required to have a high refractive index. The optical devices, such as mirrors, optical sensors, waveguide-based optical circuits, optical interference filters and solar cells, demand high refractive index materials [244,245]. In the case of polymer systems, a high refractive index is required as well as light weight, high flexibility and formability. There are many applications of high refractive index-based polymer materials, for instance, optical data storing [246], lenses [247], anti-reflective coatings [248] and immersion lithography [249]. The polymers *n* values can be improved by inserting greatly polarizable atoms or groups. The heavy atoms, except the normal atoms (C, H, O and N), usually exhibit larger polarizability, which leads to a substantial enhancement in the polymers refractive index. In fact, many polymers of high *n* value contain several elements that were documented. Herein, halogen elements, in particular, iodine and bromine, were employed as components for manufacturing polymer materials with high *n*. For example, polymethacrylates array including iodinated carbazole rings and lateral brominates were recorded (Figure 25) [250]. It is worth mentioning that the *n* value of the polymers varies in the range between 1.67 and 1.77 relies on the halogen substituent’s number and the methylene spacer’s length. However, the halogenated compounds have often shown instability to light or under some specific conditions [251].

To achieve high refractive index materials, several methodologies have been implemented. These methodologies are categorized into two main groups; incorporation of heavy metal atoms, such as sulfur and/or halogen into the polymer matrices [252] and polymer composite fabrication with high refractive index from inorganic and metal NPs [253,254,255,256,257,258]. In both methodologies, there are challenges that limit this effort to increase refractive index. In the first methodology, practical difficulty and cost are the two obstacles to insert heavy atoms into polymer matrices. For example, the achievable refractive index is 1.84 in synthesizing poly (sulfur-random-(1,3,5-triisopropenylbenzene)) [259]. On the other hand, the second methodology, there are possibility of fabricating various NPs as fillers, such as zirconium oxide (ZrO_2_) [253], titanium oxide (TiO_2_) [254,260], alumina oxide [255], gold [256] and nanodiamond [257,258]. However, two main problems have to be taken into considerations; which are relatively high surface energy and poor compatibility with polymer backbones, resulting in inorganic NPs agglomeration during insertion. Alternatively, a higher amount of inorganic NPs can be involved in polymer composite by the in situ polymerization of polymerizable monomers in the presence of inorganic NPs [261,262], or the in situ sol–gel reaction of inorganic precursors in the existence of polymer matrices [263,264].

**Figure 26:**
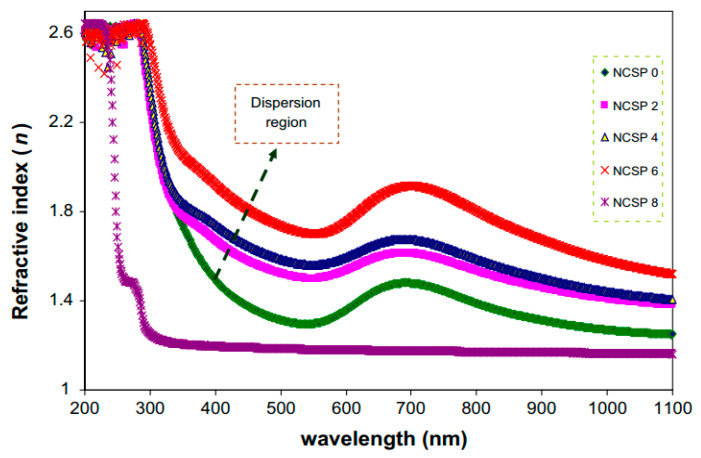
Pure and doped PMMA refractive index. The samples were symbolized as NCSP0, NCSP2, NCSP4, NCSP6 and NCSP8 for PMMA with 0 wt%, PMMA with 2 wt%, PMMA with 4 wt%, PMMA with 6 wt% and PMMA with 8 wt% of CuS, correspondingly [139].

Aziz et al. [139] studied in situ synthesis of CuS NPs in PMMA polymer matrices at ambient temperature. During characterizations of the NPs, the refractive index increment was noticed for the films including NPs of CuS. Obviously, a direct relation between the fraction of volume and the refractive index has been recorded. Interestingly, the observation of SPR peak in the spectra of the refractive index is ascribed to the formation of CuS NPs. From this study, the pure and doped PMMA refractive index spectra with different percentages (0 wt%, 2 wt%, 4 wt%, 6 wt% with 8 wt%) of CuS NPs are compared and indicated in Figure 26. It is evidently observed that the increase in the refractive index value is correlated to raising the quantity of CuS NPs. In addition, a range of the refractive index values are obtained for all the doped films in the wavelength range from 320 to 520 nm. Indeed, this increase in density resulted from rising the quantity of CuS NPs. Importantly, in the range between 570 and 980 nm wavelength a characteristic peak appears for incorporated samples. These peak outcomes of the interaction of the incident light within the range between 570 and 980 nm with the CuS NPs surface plasmons. Moreover, an increase in intensity of the peak is observed with raising the CuS amount. These results confirmed the CuS NPs creation in the films [139].

### 5.2. Dielectric Studies

The dielectric property is one of intrinsic properties of materials that represented by a complex dielectric function (ε*). It measures the response of materials during interacting with an electric field resulting in polarization. The real part of the dielectric constant in dielectric spectra represents the capability of materials to lower the speed of light within the materials. This ability originates from the extent of stored energy within the materials. The imaginary part reflects the ability of materials to take energy from the electric field as a result of dipole motion. From the real and imaginary parts, one can extract the magnitude of dielectric constant using the following relationship [265]:

(11) $$\varepsilon =\;\varepsilon_{1}+\;i\varepsilon_{2}$$

where ***ɛ*_1_** is the real part and ***ɛ*_2_** is the imaginary part of this equation which both quantities are frequency dependent.

The dielectric constants are strongly related to the permittivity of the materials. Fundamentally, permittivity is the ratio of the dielectric material permittivity to the permittivity of a vacuum. From physics point of view, permittivity means the larger the polarization produced by materials in an applied field of a given strength, signifying the larger value of the dielectric constant [266].

The electrolyte of choice in energy storage devices is polymer materials because of low cost, low dielectric loss and light weight. In fact, the polymer materials to be applicable in these devices; need to have a high capacitance density which will be achieved with high dielectric constant. Recently, many efforts have been devoted to modify the dielectric property of polymer materials to match the requirements to be embedded in capacitors. To be eligible for application in energy storage devices, many conductive polymer composites have been designed [267,268,269]. Of special interest, NPs can be added to the host polymer to enhance the dielectric properties. These materials are widely usable in energy storage capacitors and in battery management systems in the microgrid which are safe and environmentally friendly [270]. The NPs have larger interfacial area per unit volume compared to the microsized fillers; therefore, these particles uniformly disperse into the polymer materials. Thus, the capacitance of polymer NCs has increased mainly as a result of the inherent permittivity of NPs, loading and wide interfacial property between polymers and NPs [271]. The composite polymer materials containing inorganic dopants exhibit dielectric constants of 100 folds larger than that of pure polymer hosts [272,273]. This is correlated to the high dielectric constant of the inorganic dopants [274]. An unsatisfactorily high rise in dielectric loss, such as energy dissipation must not be responsible for increasing the effective dielectric constant. To be clearer, it has not supposedly been achieved to see large breakdown field strength, large dielectric constant and small dielectric loss at the same time instead a compromise will occur. As a consequence, focus is being directed to develop polymer composite materials through a superior understanding of the physical phenomena that govern both breakdown field strength and dielectric permittivity. These two phenomena are usually involved in the region of interface between the fillers and polymers. Therefore, better understanding of the chemistry of the fillers and polymers are the priority task of research in this field. It is of effective way to add ferroelectric metal oxides such as Pb(Mg_0.33_Nb_0.77_)O_3_-PbTiO_3_ (PMNT), Pb(Zr,Ti)O_3_ (PZT) and BaTiO_3_ (BT) into dielectric polymer composites to enhance the dielectric permittivity. From an effective dielectric constant point of view, the inorganic fillers insertion with dielectric constants approximately hundreds and thousands is an appealing way to increase polymer composites. It is known that the polymers mostly possess dielectric constants smaller than 10 [275]. However, this high dielectric constant of dopants (fillers) results in local electric field at the interfacial region between the dopants and polymer bodies. This electric field could be larger than the external electric fields, causing substantial lowering of the electric breakdown strength [276]. This is considered as a significant drawback of these polymer composite materials to be utilized at the large scale. Therefore, nanostructure engineering has been implemented in an attempt to lower the interfacial effect [276]. Thakur et al. [274] recorded a substantial rise in the dielectric behavior which is obtained for polyetherimide NCs with NFs whose dielectric constant is analogous to that of the host polymer. These NFs can lower the constraint on the response of dipole toward the used electric field; therefore, increasing the dielectric constant achieved. Alternatively, via nanostructure engineering, one can profoundly improve the dielectric constant of NCs even in the absence of high dielectric constant NFs.

The dielectric property is one of optical properties of solid materials [243]. Principally, the dielectric constant is a strongly photon energy dependent property. To explain this, there are interactions between electrons and photons in the film which lie within an energy range. The nature of the interactions determines the main feature of the peaks in the dielectric spectra [241]. Moreover, refractive index and extinction coefficient are also effective and mathematically expressed as follow [118,277]:

(12) $$\varepsilon_{1}=\;n^{2}-\;k^{2}=\;\varepsilon_{\infty }-\;\frac{e^{2}}{4\pi C^{2}\varepsilon_{0}}\frac{N}{m^{*}}\lambda^{2}$$

(13) $$\varepsilon_{2}=2nk$$

where the dielectric constant at highest wavelengths is symbolized by ***ɛ_∞_***, the vacuum dielectric constant is symbolized by ***ɛ_o_***, the localized electronic state density to effective mass ratio is indicated by$\;\frac{N}{m^{*}}$; *e* and *c* have usual meanings [118].

**Figure 27:**
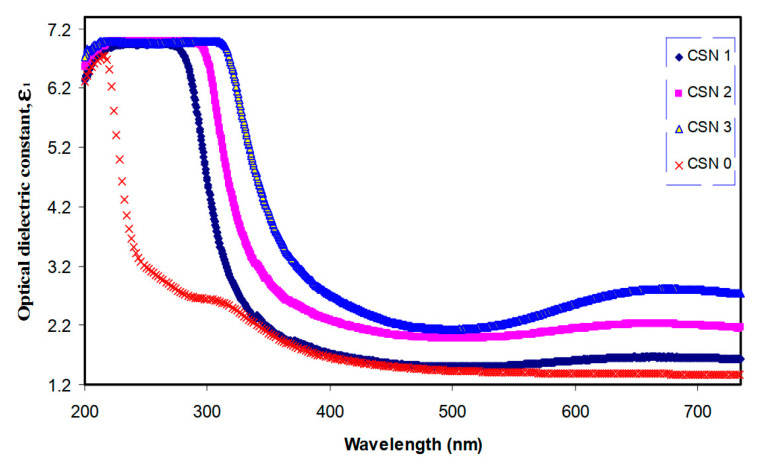
Optical dielectric constant spectra against wavelength for pure CS and doped CS films. The chitosan nanocomposite (CSN) films were symbolized as CSN0, CSN1, CSN2 and CSN3 for CS doped with 0 wt%, 4 wt%, 8 wt% and 12 wt% CuI, correspondingly [78].

S.B. Aziz [78] synthesized copper (Cu) NPs using in situ methodology with noticeable surface plasmonic resonance (SPR) peaks. In this synthesis, chitosan host polymer acted as both a capping agent and a reducing medium for the CuNPs. Figure 27 illustrates the optical dielectric constant (***ε*_1_**) versus wavelength. It is seen that upon incorporation of various quantities of CuI (0 wt%, 4 wt%, 8 wt% and 12 wt%), a profound increase in dielectric constant is observed. This growth can be described based on increasing the density of states because *ε*_1_ is directly correlated to the density of states within the solid polymer films forbidden gap [66]. In other words, an increment in dielectric constant indicates introducing extra species carriers to the host material body; as a consequence, an increase in the density of states arises [78,144].

### 5.3. Band Gap Study

Band gap energy is an energy range with no electron states in solid materials [278]. These states lie between the valence and conduction bands which normally prohibit any transition; in other words, it has forbidden energy. To make a transition from these two bands, it is necessary to have sufficient energy which equals the forbidden energy. As a consequence, electrons are able to move freely as mobile charge carriers between the valence and conduction bands [279]. Generally, there are energy band gaps of insulators and semiconductors which are 10 eV and ~1 eV, respectively. Contrast, conductors possess an overlapping of the valence and conduction bands. A comprehensive understanding of the nature of the electron transition in semiconducting polymers and charge transfer complexes has not been established yet. In order to have electrons transfer from valence to conduction bands, the incident photons need to have sufficient energy to overcome the forbidden energy and the phonons must provide the required momentum [280].

**Figure 28:**
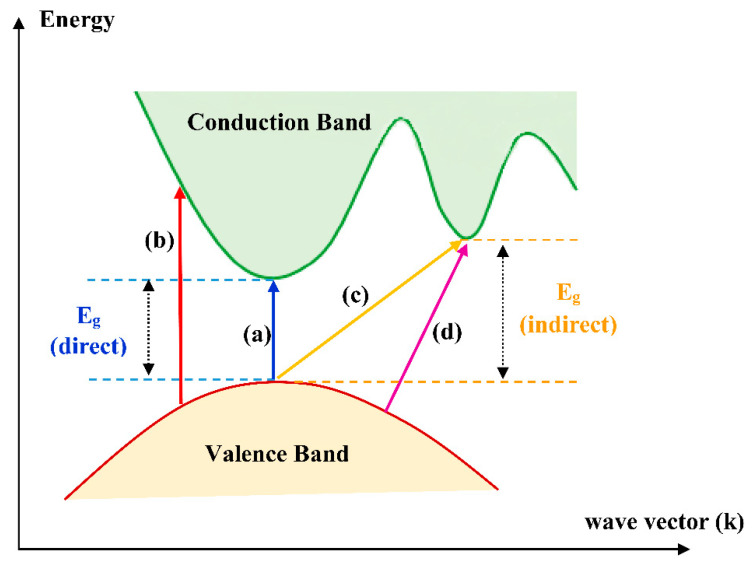
Electronic transition (**a**) allowed direct, (**b**) forbidden direct, (**c**) allowed indirect and (**d**) forbidden indirect [303].

The previous published studies have verified that the band gap energy can be estimated on the basis of dielectric loss, and the type of electron transition has specified using Tauc’s model [78,116,144,281,282,283,284,285,286]. It is facile to estimate the optical dielectric function and predict the extent of overlapping bands within materials using UV–vis spectroscopy. Meanwhile, the linear response of materials toward electromagnetic radiations as optical property can be understood through the complex dielectric function $\varepsilon^{*}=\;\varepsilon_{1}-i\varepsilon_{2}$ [287]. Based on a study, there is a strong relationship between the valence (occupied) and conduction (unoccupied) bands [281]. To correlate the optical absorption of materials to the imaginary part ***ε*_2_**, it is straightforward mathematically as shown in the following complex equation [118]:

(14) $$\varepsilon_{2}\left(\omega \right)=\;\frac{2e^{2}\pi }{\Omega \varepsilon_{0}}\underset{K,V,C}{\sum }|\Psi_{K}^{C}\left|\vec{U}.\vec{r}\right|\Psi_{K}^{V}{|}^{2}\delta \left(E_{K}^{C}-\;E_{K}^{V}-\;ћ\omega \right)$$

where ***ω*** and ***Ω*** represents the incident photon and the volume of the crystal, respectively and the vacuum permittivity and charge of electron are represented by ***ɛ_o_*** and ***e***, respectively. It is also shown that the incident electromagnetic wave polarization vector and position vector are expressed by$\vec{\;U}$ and $\vec{r\;}$, respectively. Finally, the wave functions conduction and valence bands at *K* are symbolized as $\Psi_{k}^{v}$ and $\Psi_{k}^{c}$, respectively. The theoretical principle of optical dielectric constant is based on a complex function of frequency. Thus, large-scale computational endeavor is needed to calculate dielectric constants [288,289,290,291]. On the other hand, experimental estimation of dielectric constant from the optical dielectric function on imaginary part (***ε*_2_**) is easily achieved using refractive index and extinction coefficient in the calculation as shown in the following relationship [78,281,282,283,284,285,286]:

(15) $$\varepsilon_{2}=2nk$$

where ***K*** and ***n*** are the extinction coefficient and refractive index, respectively. Previously, it has been proved that there are interband transitions from the appearance of the new peaks in the optical dielectric loss (***ɛ_i_***) spectra [291,292,293,294,295,296]. Then, the band gap energy can be determined from the intersection of the optical dielectric loss linear part with the axis of the photon energy in the spectra. This can be explained from this strong relationship between the optical dielectric function and the consequence of electron-photon interaction. This relationship indicates the interband transitions within the electronic state of solids. The response of the transitions of electrons from occupied to unoccupied states determines the dielectric function of the imaginary part (***ε_i_***) [285,295]. It has been previously validated that the cause of the imaginary part is these transitions [291]. Herein, it is of great importance that electron excitation from the valence band to the empty conduction band resulting from photon absorption is called interband transition. The mechanism involves absorption of photons, creating excited states and leaving holes behind is explained on the basis of quantum mechanical perspective [296]. It is also stated from quantum mechanics (microscopic) that a strong relationship between the electronic states and the optical loss parameter exists depending on whether they are filled and unfilled in solids. As previously mentioned there is also a correlation between the imaginary part of the dielectric function and interband transitions that has been confirmed from quantum mechanics [285,288,295]. Additionally, it is of great importance to take advantage of optically induced transition and band structure of materials from analysis of optical absorption process [297]. Quantum mechanics establishes selection rules that govern the transitions from one state to another [298,299]. Hence, different types of transition, depending on the nature of band structure have been counted [299]. To exceed the band gap energy gap (***E_g_***) in an attempt to make the transition of electrons from the valence to conduction bands, it is necessary to have enough optical energy to be absorbed. The absorption of energy to make an electron transition from the valence to conduction bands is equivalent to the band gap energy. The mathematical expression of the energy absorbed (***hυ***) that exceeds the fundamental absorption edge is shown below [300,301]:

(16) $$\alpha h\upsilon =B{\left(h\upsilon -\;E_{g}\right)}^{\gamma }$$

where ***B*** stands for a constant related to the band tailing extent, and ***hυ*** stands for the incident photon energy. The coefficient of power symbolized by ***γ***. It is computed based on the kinds of possible electronic transitions, i.e., 1/2 for direct allowed while 3/2 for direct forbidden, two for indirect allowed and three for indirect forbidden [281,285]. Figure 28 illustrates the different types of electronics transition that can exist between the valence band and conduction band based on Tauc’s model [302].

Based on the solid-state physics view, in solid materials, there are energy ranges that represent energy gaps where the energy levels are not allowed. This means there is an absence of absorption of free charge carriers and that the action of interband transitions is limited to cases of photons with high energies [284].

Abdul-Kader [304] studied optical band gaps via Tauc’ model analysis. For the study, ultrahigh molecular weight polyethylene (UHMWPE) was irradiated by a beam of electrons. A 1.5 MeV electron beam with a dose range between 50 and 500 kGy was applied in the irradiation process. The study confirmed that the optical absorption of all irradiated samples can be increased compared to the untreated ones. This increment in electron beam absorption during irradiation might be due to the new electronic states’ formation within the forbidden gap [305]. It is observed that pristine UHMWPE possesses a band gap of 3.25 eV whereas with irradiated of 500 kGy, the band gap lowers to 2.7 eV. This energy gap decrement can be associated with the formation of clusters enriched in carbon as a result of releasing hydrogen as hydrogen gas [306,307]. It is also possible to create intermediate energy states from rearrangements of structure [308]. Thus, lowering the band gap energy does not only result in increasing electrical conductivity, but also enhances the optical properties of the irradiated polymers [304,309]. It has also shown that exponent ***γ*** takes 1/2 and 3/2 values for the direct allowed and forbidden transitions, respectively. For indirect allowed and forbidden transitions, the exponent ***γ*** takes the two and three values, respectively.

In the study, the authors took only ***γ*** = 1/2 into consideration and ignored the rest values of the exponent. The proved outcomes have shown that the allowed direct transition (***γ*** = 1/2) indicates the vertical electron transitions from the top of the valence band to the bottom of the conduction band whereas the nonvertical transitions (indirect transitions) are impossible [304,310]. Shehap and Dana [154] synthesized polymeric-films-based polyvinyl alcohol (PVA) that incorporated with various quantity of titanium dioxide nanoparticles (TiO_2_/PVA): 1.25, 2.5, 5, 7.5, 10 wt% using casting techniques with the aid of sonification. It was shown that the optical band gap was reduced with the addition of TiO_2_ contents. However, the authors had not specified the type of electronic transition within pure PVA. It was found that the value of ***E_g_*** of PVA pure was 4.8 eV which is nearly comparable to that reported 5.1 eV [311] and 5.05 eV [312]. It was also seen that the value of ***E_g_*** for pure PVA was higher than all the composites and it declined with rising TiO_2_ NPs in the host matrix of PVA, indicating defect formation (extra energy levels). These imperfections could produce localized levels within the optical band gap that increase with an increasing dispersion of the imperfections [154].

It is worth mentioning that using alkali metals such as (KCl, LiI, NaI, KI, RbI and CsI) would not result in a significant reduction in the value of the band gap of the host polymers [73,313]. These alkali salts reduced the band gap of host polymers like PVA and PEO very slightly which does not meet the expectation [73,313]. Yassin et al. [229] used a modified Hammer’s method in the synthesis of graphene oxide from graphite oxide nanopowder with an oxidation degree within 5–10%. The incorporation of the various quantities of 0.2, 0.4, 0.8, 1.6, 3 and 4 wt% of graphene oxide (GO) into poly (Vinyl chloride-co-vinyl acetate-co-2-hydroxypropyl acrylate) (PVVH) copolymer have been successfully carried out. It was shown that the decrease in ***E_g_*** with increasing the filler caused by several polaronic and imperfection formation within the matrices. However, the type of electronic transition has not been specified and they mentioned that for the direct band gap, the energy gap was reduced from 4 to 3.7 for the pure PVVH and 4 wt% of GO, respectively. Furthermore, for the indirect band gap, energy gap was reduced from 3.55 to 2.5 eV for the pure PVVH and 4 wt% of GO, respectively [229].

Unfortunately, the specification of the type of electronic transitions has not been documented in the literature frequently [314,315,316,317,318]. In the determination of the band gap and specifying the electron transition from the top of the valence band to the bottom of the conduction band, both the optical dielectric loss parameter and Tauc’s model must be taken into consideration. In recent publications, the optical dielectric loss parameter and Tauc’s model have been successfully used in the study of bandgap and the type of electronic transitions [78,116,144,281,285,286,319]. These recently published works can be used as an advocate to the hypothesis that both the optical dielectric loss parameter and Tauc’s model are satisfactory to tackle the band gap and electron transition types, correspondingly. 

Aziz et al. [191] used hydrothermal treatment in the synthesis of CNDs using glucose as well as inserted the CNDs to PVA to create incorporated PVA with 0 mL, 15 mL and 30 mL of 5 mg of dissolved CNDs by solution casting technique. The incredible observation is the band gap reduction of CNDs inclusion to PVA from 6.2 to 5.12 eV. More observation was noted, as CND portions were increased, the value of ***E_g_*** decreased which might result from the formation of a larger number of free species carriers/radicals [320]. As a consequence, these composites can be used for various applications, such as optoelectronics [191]. There are controversial topics about the main effect of band gap energy on the dielectric constant within the wavelength range [288]. Penn in 1962 [321], established a model that correlates between energy band gap and optical dielectric constant [118,289,321].

(17) $$\varepsilon_{\left(0\right)}\;\approx 1+\;{\left(\frac{ћ\omega_{p}}{E_{0}}\right)}^{2}$$

**Figure 29:**
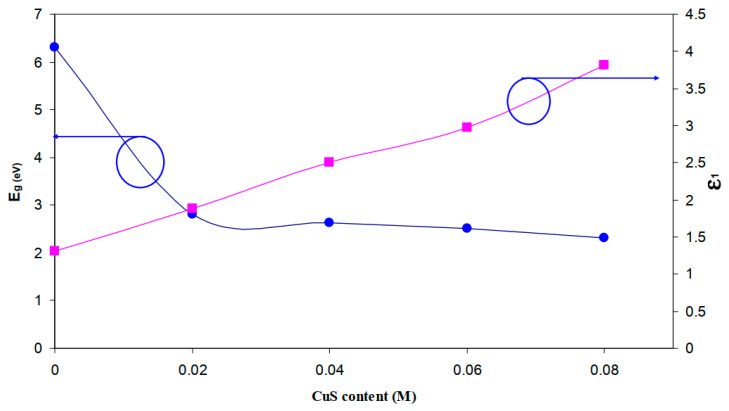
Energy bandgap and optical dielectric constant against amounts of CuS [281].

This model relates the refractive index (***n***) to the dielectric constant in the form of ***ε*** = ***n*** [322]. According to this model, dielectric constant keeps constant over a long wavelength range that can be used to estimate energy states [290]. Consequently, as the optical dielectric constant increases, the density of states increases and the band gap energy drops. Three main factors; design, the method of synthesis and the proper choice of the polymer of small band gap usually lower than 2 eV determine the eligibility of organic photovoltaics to be utilized in solar cells [323]. The model summarily and descriptively states that energy band gap reduces as the density states increase [118]. Aziz et al. [281] used sodium sulfide (Na_2_S) and copper chloride (CuCl_2_) salts in the copper monosulfide (CuS) NPs fabrication and then incorporation of the NPs into methylcellulose (MC) to fabricate small optical band gaps of polymer NCs. Figure 29 shows the energy bandgap and optical dielectric constant against the CuS dopant. From Figure 29, it is deceptive that the ***ɛ*_1_** largest value is obtained at the minimum optical bandgap value. The previous correlations confirm the validity of Penn’s model. It is shown experimentally that the optical bandgap along with the electronic structure are simply tackled by the optical dielectric function [281].

**Figure 30:**
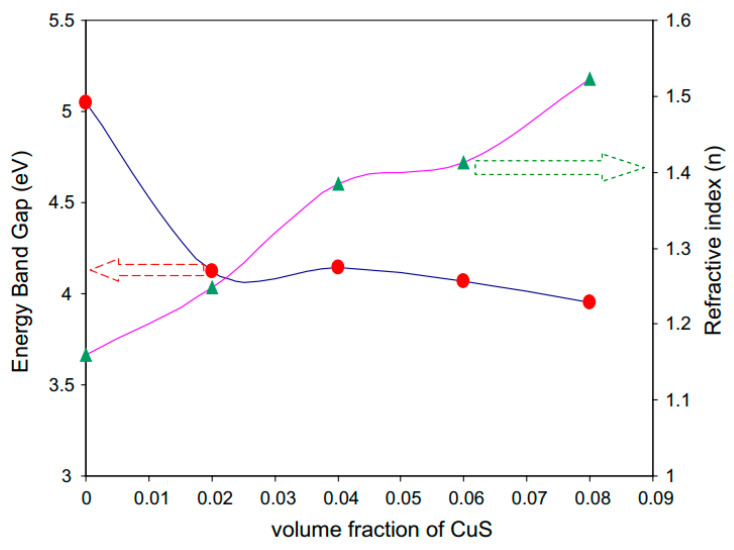
Energy band gap and refractive index (at 390 nm) of PMMA against CuS nanoparticles [139].

Aziz et al. [139] synthesized CuS NPs in situ and then PMMA polymer incorporated with CuS NPs at ambient temperature. The establishment of an inverse proportionality between band gap energy and the refractive index has been achieved as shown in Figure 30. This shows the correlation between these two parameters in the presence of 0, 2, 4, 6 and 8 wt% of CuS NPs. It was found that the addition of the CuS NPs changed the general trend of the relationship between the band gap and the refractive index. The optical properties; band gap and refractive index relationship on one side and the impact of chemical composition and atomic arrangements on the other side have been well established [324]. It is of impressive interest that the creation of localized states due to the addition of dopants in PMMA and the PVA band gap have been well documented [51,139].

The illustration of the influence of CuNPs, copper powder, and copper complex in the reduction of energy gap is of crucial importance. Aziz et al. [85] studied and synthesized solid polymer composites (SPCs) based on polystyrene using a solution cast technique. In the study, copper (Cu) powder was added from 0 to 6 wt% in the synthesis of samples. Here, an interesting observation was upon the addition of Cu content, the energy gap reduced. It was concluded that the band gap’s energy reduced certainly at 6 wt% of Cu powder. To interpret this observation, it is believed that powder of Cu inserts multiple valence states into the structure of polystyrene and consequently reduction in the band gap energy between the valence and conduction bands occurs. Of special interest, within the inclusion of Cu powder, the energy gap reduced from 4.05 to about 3.65 eV could be ascribed to the introduction of multiple states into the structure [85].

Aziz [78] used sodium sulfide (Na_2_S) and copper chloride (CuCl_2_) salts in the copper monosulfide (CuS) NPs fabrication. In the study, various portions of CuS NPs; 0, 0.02, 0.04, 0.06 and 0.08 M were inserted into methylcellulose (MC) polymer to fabricate small energy band gaps polymer NCs. The copper (Cu) NPs were fabricated by an in situ method with considerable surface plasmonic resonance (SPR) peaks. The methylcellulose (MC) host polymer has dual functions; as a reducing medium as well as a capping agent for the CuNPs [78]. It was noticed that the MC optical band gap energy has been decreased from 6.2 to 2.3 eV upon inserting 0.08 M of CuS NPs. The optical band gap energy (***E_g_***) has been determined to be lowered for the incorporated films. 

Such a reduction in band gap energy can be described based on the fact that complexes of charge transfer in the host polymer occurred, resulting from the incorporation of tiny amounts of the filler [325]. It is of great importance to enhance the low energy transitions, leading to a substantial reduction in optical band gap energy [326]. These studies indicate that functional materials, organic polymers and composites with reasonable optical band gaps are important for organic light-emitting diodes (OLED), photonics and optoelectronics devices [327,328,329,330].

Brza et al. [118] reported the synthesis of Cu(II)-complexes using the green method. From the extract of the tea, it is possible to synthesize Cu(II)-complex. The Cu metal complexes with various extracts of 0, 15, 30 and 45 mL were integrated with PVA polymer to form polymer composites with significantly reduced optical band gap energies. From the finding, it is indicated that the band gap shifted from 6.2 eV to 1.4 eV for PVA doped with 45 mL of Cu(II)-complex. It is understood that the reduction in the optical band energy with Cu(II)-complex substantially occurs compared to copper powder and copper NPs additions.

## 6. Conclusions and Perspectives

The polymer electrolytes and composites applications in numerous electrochemical, electrical and optoelectronic devices have been improved recently. Various types of polymer electrolytes have been investigated and documented in the literature. The use of composite polymer electrolytes was recognized as a likely technique to develop the characteristics of the electrolytes. Different approaches were established to fabricate the composite polymer electrolytes, such as the incorporation of metal NPs, metal complexes, semiconductor NPs, ceramic fillers, carbon NTs and graphene. The approaches to improve the optical properties of polymer electrolytes were highly emphasized. Several documents have indicated that the composite polymer electrolytes possess benefits over traditional liquid electrolytes, particularly in their safety and flexibility features. The optical parameters for example optical dielectric constant, absorption coefficient and refractive index are described. The optical band gap was investigated using two methods: Tauc’s model and the optical dielectric loss parameter. Based on recently published articles sufficient quantum mechanical backgrounds are provided for applicability of the optical dielectric loss parameter to the investigated band gap. In this review paper, it was demonstrated that both Tauc’s model and the optical dielectric loss should be studied to specify the type of electron transition and estimate the optical band gap correctly.

The intensive and extensive survey of the literature indicated that polar polymers that are cheap and less cost-effective are generally good insulators and their bandgap are wide which limits their application in photovoltaic and optoelectronic applications. Fillers and nanoparticles are not sufficient to reduce the band gap of polar polymers. Based on our recent achievements metal complexes produced by green coordination chemistry is crucial to be considered to fabricate polymer composites with desired optical band gaps. Based on experimental and theoretical approaches, we arrived at the fact that the optical dielectric loss parameters should be studied in order to specify the type of electronic transition from Tauc’s model.
